# Eps2Fold: a rapid method to characterize G-quadruplex DNA structures using single absorbance spectra

**DOI:** 10.1093/nar/gkaf953

**Published:** 2025-09-29

**Authors:** Eric Largy, Aurore Guédin, Amani Kabbara, Jean-Louis Mergny, Samir Amrane

**Affiliations:** Univ. Bordeaux, INSERM, U1212, CNRS, UMR 5320, ARNA, IECB, Bordeaux F-33000, France; Univ. Bordeaux, INSERM, U1212, CNRS, UMR 5320, ARNA, IECB, Bordeaux F-33000, France; Univ. Bordeaux, INSERM, U1212, CNRS, UMR 5320, ARNA, IECB, Bordeaux F-33000, France; Univ. Bordeaux, INSERM, U1212, CNRS, UMR 5320, ARNA, IECB, Bordeaux F-33000, France; Laboratoire d’Optique et Biosciences, Ecole Polytechnique, CNRS, INSERM, Institut Polytechnique de Paris, Palaiseau F-91120, France; Univ. Bordeaux, INSERM, U1212, CNRS, UMR 5320, ARNA, IECB, Bordeaux F-33000, France

## Abstract

G-rich oligonucleotides can fold into non-canonical secondary structures called G-quadruplexes (G4s), consisting of stacked G-tetrads, i.e. planar arrays of four guanines connected by eight hydrogen bonds and coordinating cations such as K^+^ and Na^+^. G4s are remarkably polymorphic structures, which enables them to participate in various biological processes and find applications in nanotechnology. The development of simple and rapid assays to better understand how and when G4s form remains very important. Here, we present Eps2Fold (Epsilon-to-Fold), a method for detection and characterization of G4 conformations by simply measuring a single UV-absorbance spectrum. Eps2Fold is conceptually similar to isothermal differential spectra or thermal differential spectra but is generated by the subtraction of an experimentally recorded UV absorbance spectrum of the G4 in its folded state and a calculated UV absorbance spectrum that mimics the G4 in its unfolded state. Through comprehensive biophysical and principal component analysis (PCA) analysis of thirty G4-forming oligonucleotides, we demonstrate that Eps2Fold provides spectral signatures with unexpectedly high structural resolution remarkably similar to that obtained with circular dichroism spectra. Both techniques allow precise topology assignment based on the number and composition of stacked *syn* or *anti* guanines glycosidic bond angle stacks. The spectral differences between conformers are subtle and best analyzed using multivariate statistical approaches such as PCA rather than visual inspection alone. We provide an open-source GUI program to facilitate such analysis. Users simply upload the UV absorbance spectrum of the G4 on the web application (https://github.com/EricLarG4/Eps2Fold); structural analysis will run automatically. Eps2Fold represents a rapid and inexpensive structural approach enabling precise characterization of G4 structures from a single UV absorbance spectrum.

## Graphical Abstract

**Graphical Abstract:**
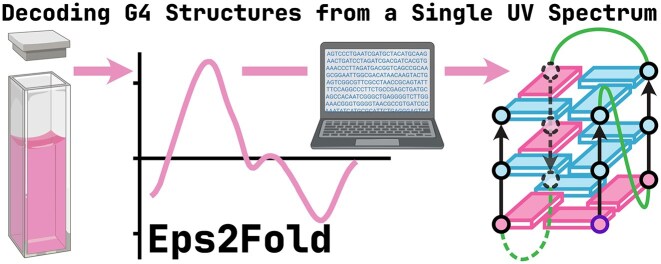

## Introduction

G-rich sequences of nucleic acids can fold into non-canonical secondary structures called G-quadruplexes (G4s) [1, 2]. G4s are involved in the regulation of a number of biological processes at the DNA and RNA levels [3–9]. G4s are quadruple helices markedly different from the classical B-DNA double helix, and these structural differences make them attractive targets for small molecule therapeutics. G4s are also found in many aptamers and constitute promising building blocks for nanotechnology applications [10–15]. To better understand how and when G4s form, how to target them specifically, and how to direct their folding, it is necessary to characterize their structure.

**Figure 1.**
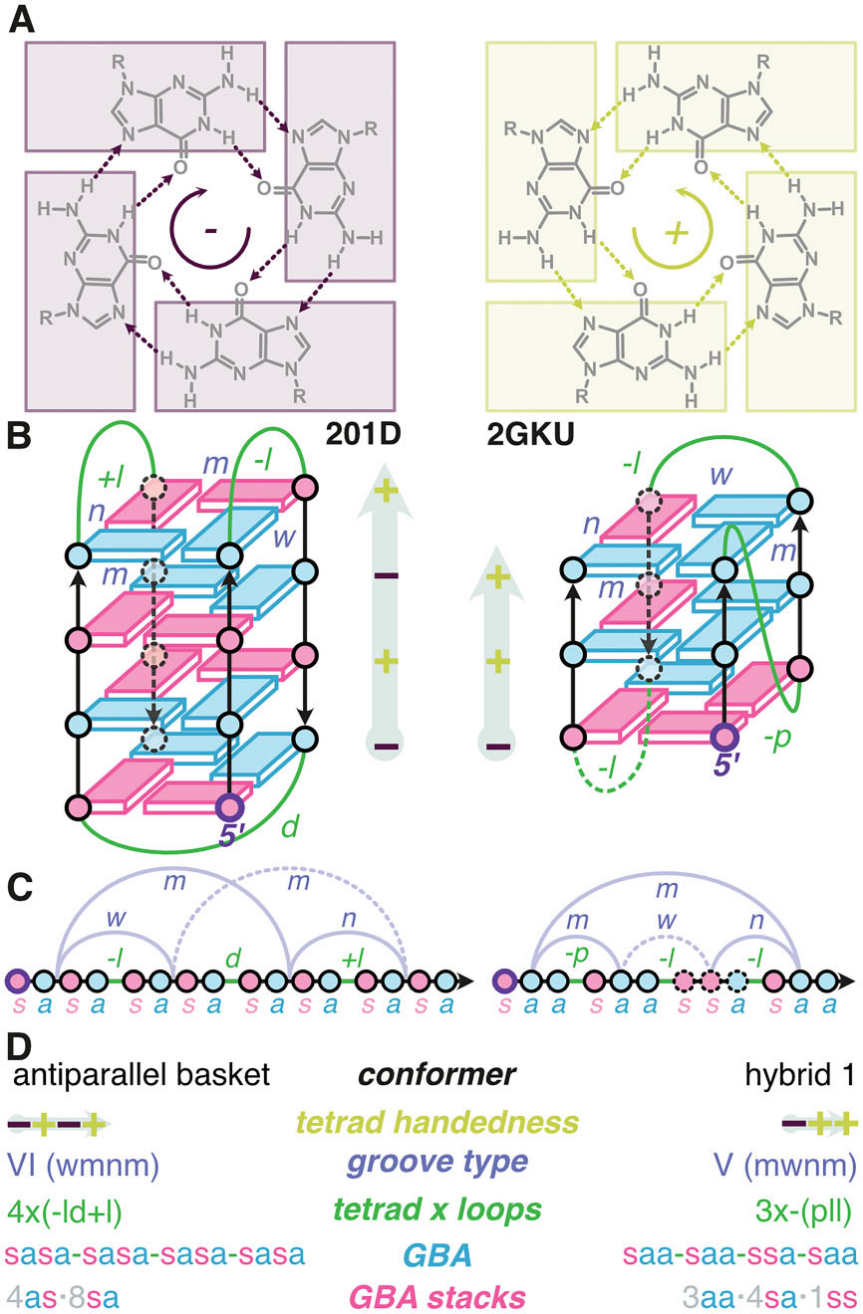
(**A**) Guanine tetrads and their handedness, defined by the H-bond donors-to-acceptors direction, either clockwise (–) or counterclockwise (+). (**B**) Schemes of antiparallel basket (pdb 201D) and hybrid 1 (pdb 2GKU) topologies. Guanines involved in tetrads are depicted as rectangles capped with a circle on the G-tracts (black arrows), which are connected by green loops. Blue and pink guanines have *anti* and *syn* glycosidic bond angles (GBAs), respectively. Tetrads’ handedness are + or – following the definition of panel (A), and collected looking from the top and starting in 5′. Loops have propeller (p), lateral (l), or diagonal (d) geometries. Loops are assigned a + or – when linking guanines in a clockwise or counterclockwise fashion, respectively, with the 5′-end positioned at the bottom right of the structure. Diagonal loops (d) have no + or – assignments. Grooves are either wide (w), medium (m), or narrow (n). (**C**) Linear representations of the schemes from panel (B). (**D**). Summary of the six structural descriptors of the topologies 201D and 2GKU from panel (B).

However, there is no unique G4 structure but a wide variety of conformers. A single G-rich sequence, in a given set of experimental conditions, can often form several conformers in equilibrium [16, 17]. All G4s, though, share some key structural elements. Minimally, a G4 is generally, but not always [18], formed by (i) the stacking of at least two tetrads (also called G-quartets), themselves composed of four co-planar guanines linked by eight hydrogen bonds linking their Watson–Crick and Hoogsteen faces (Fig. 1A), and (ii) the coordination of a cation by the guanine O6 within each pair of consecutive tetrads. The most relevant cations in a biological environment are K^+^ and Na^+^, but others like NH_4_^+^, Rb^+^, Sr^2+^, or Ca^2+^ can also promote the formation of G4s [16, 19]. However, cations with too small (Li^+^) or large (Cs^+^) atomic radii cannot.

Many structural elements can vary between two G4 conformers: the number of tetrads, the relative orientation (relative to the 5′ to 3′ polarity) of the four guanines strands, or G-tracts, the geometry of the loops (propeller, lateral, diagonal), and the Glycosidic Bond Angles (*syn or anti*GBAs) of tetrad guanines [20]. Additional interactions including base pairs, triads, or non-G-tetrads may also be involved. Historically, G4s have been classified in families of topologies: parallel (all G-tracts in the same 5′-3′ orientation) or antiparallel (two strands in one direction and two strands in the opposite direction; Fig. 1B), to which the hybrid category was added in 2006 (three strands in one direction, one in the opposite; Fig. 1B) [21]. The antiparallel class may be subdivided into the chair (parallel strands are diagonally opposed, all three loops are lateral) and basket conformations (the parallel strands are adjacent to each other and contain a diagonal loop and two lateral loops) [22]. It is in the opinion of the authors, and others, that classifying G4s based on these topologies is not sufficient to capture the diversity of G4s: other structural descriptors (Fig. 1C and D and “Materials and Methods” section) have been proposed such as tetrad handedness, groove type combination, tetrad loop progression, and GBAs composition [20, 23–25]. Here, we also introduce the GBA-stacks combination (Fig. 1D).

The determination of structures by high-resolution techniques is very informative but can be very challenging and time-consuming. When several sequences must be quickly characterized, lower-resolution techniques are used. Circular dichroism (CD) is routinely used to confirm the folding of G4s and assign a topology by comparison with typical signatures [22, 26–29]. Thermal difference spectra (TDS), obtained by the subtraction of UV spectra acquired at low (folded state) and high (unfolded state) temperatures, is an alternative method to obtain characteristic spectral signatures for a variety of secondary structures without the need for a specialized instrument. It is, however, not an isothermal method, which limits its use for e.g. folding and binding kinetics [16, 30]. In addition, obtaining a reliable TDS may be challenging if the structure is thermally stable, as often found for G-rich RNA samples.

Isothermal difference spectra (IDS) is a variant of TDS that does not rely on high temperature to unfold nucleic acids [31]. Rather, it is often calculated by subtracting spectra obtained in the absence and presence of a cation promoting their folding. It is therefore only applicable to structures requiring the presence of specific cations to fold, which is the case of G4s. In specific cases for which folding/unfolding is slow enough (e.g. intermolecular G4s), the kinetic inertia of the system may allow recording the absorbance spectra of the folded and unfolded forms at the same temperature and under identical ionic conditions. This is the case, e.g. after fast cooling of a denatured G4: if refolding at low temperature is slow enough (minutes to hours scale), then an absorbance spectra taken immediately after cooling reflects a mostly denatured sample, while the same spectra recorded after overnight incubation would correspond to a mostly renatured sample. In that case, even if folding or unfolding is incomplete, the shape of the TDS or IDS is sufficient to determine the structure.

IDS is used significantly less frequently than CD and TDS, and even less so for the purpose of determining topologies. Yet, we have already presented anecdotal evidence that IDS can discriminate between G-quadruplexes of different topologies (parallel and antiparallel) [16]. Obtaining an IDS signature using a G4-promoting cation like K^+^ (versus a reference in e.g. Li^+^) is therefore an elegant orthogonal approach to CD to demonstrate the formation of a G4 with a simple UV-vis spectrophotometer. A major limitation of IDS is the experimental challenge of obtaining an absorbance spectrum in the absence of G4-promoting cations, even after extensive desalting of the oligonucleotides. In fact, stable G4s can be formed in stoichiometric, micromolar amounts of potassium [32], and therefore may still be partially folded after desalting. This results in claims in the literature that some G-rich sequences can fold into solutions devoid of G4-forming cations and, more importantly in the context of this manuscript, in inaccurate unfolded-state UV spectra and in turn in IDS with lower intensities than expected [31].

Here, we propose a new method, coined Eps2Fold (Epsilon-to-Fold), which provides insights into G4 conformations. This approach is analogous to TDS and IDS (i.e. subtraction of folded and unfolded state spectra), yet it relies on the measurement of a single folded state spectrum. In Eps2Fold, the unfolded state spectrum is calculated from the oligonucleotide sequence (see “Materials and methods” section), as opposed to being measured experimentally. Eps2Fold’s primary advantages over IDS are twofold: first, it circumvents the issues associated with the production of unfolded G-rich oligonucleotide solutions, and second, it expedites the analytical workflow.

The objective of this manuscript is to assess the Eps2Fold approach through a thorough biophysical and principal component analysis (PCA) of a reference set of thirty G4-forming oligonucleotides including one left-handed Z-G4 structure (Table 1) [20, 33–55]. We found that, similarly to CD, Eps2Fold signatures are mainly governed by the stacking geometry of guanines from the G4 core, and sometimes from loops, rather than the strand orientation (parallel, antiparallel, or hybrid) classically used to define topologies. We found that the GBA-stack G4 descriptor is the best suited to interpret CD and Eps2Fold signatures. As a result, both Eps2Fold and CD spectra allow to assign G4 conformations according to their composition in GBA-stacks, i.e. the total number of 5′-*syn*/*anti*’-3′ (sa), 5′-*anti*/*syn*-3′ (as), *5′-syn/syn-3′* (ss), and *5′-anti/anti-3′* (aa) steps.

**Table 1.** Sequences and structural descriptions of the reference panel

Name	Sequence	Salt	Conformer	Grooves	Tetrad × Loop	Groove combination	Handedness	GBA	GBA stacks	Ref.
Bom-U16	TAGGTTAGGTTAGGTTAGG	K^+^	Antiparallel chair	wnwn	2×−(lll)	II	-+	(sa)4	4sa	[33]
148D	GGTTGGTGTGGTTGG	K^+^	Antiparallel chair	wnwn	2×+(lll)	II	-+	(sa)4	4sa	[34]
Hivpro1	TGGCCTGGGCGGGACTGGG	K^+^	Antiparallel chair	wnwn	2×−(lll)	II	-+	(sa)4	4sa	[35]
2KM3	AGGGCTAGGGCTAGGGCTAGGG	K^+^	Antiparallel chair	wnwn	2×−(lll)	II	-+	(sa)4	4sa	[36]
5YEY	GGGTTAGGGTTAGGGTTTGGG	K^+^	Antiparallel chair	wnwn	3×+(lll)	II	-++	saa-ssa-saa-ssa	2aa·4sa·2ss	[37]
5J4W	GGTTTGGTTTTGGTTGG	Na^+^	Antiparallel basket	wmnm	2×(−ld + l)	VI	-+	(sa)4	4sa	[20]
5J4P	GGTTTGGTTTTGGTTTGG	Na^+^	Antiparallel basket	wmnm	2×(−ld + l)	VI	-+	(sa)4	4sa	[20]
2M6V	GGGTTGGGTTTTGGGTGGG	Na^+^	Antiparallel basket	wmnm	2×(−ld + l)	VI	-+	(sa)4	4sa	[20]
6GZN	GGGTAGGGAGCGGGAGAGGG	K^+^	Antiparallel basket	wmnm	2×(−ld + l)	VI	[-]-+[+]	sa[a]-sa[a-a]sa-sa[a]	[3aa·1as]·4sa	[38]
5J05	GGGTTTGGGTTTTGGGAGGG	Na^+^	Antiparallel basket	wmnm	3×(−ld + l)	VI	-+-	sas-asa-sas-asa	4as·4sa	[20]
143D	AGGGTTAGGGTTAGGGTTAGGG	Na^+^	Antiparallel basket	wmnm	3×(−ld + l)	VI	+-+	asa-sas-asa-sas	4as·4sa	[39]
2MFT	GGGTTTTGGGTGGGTTTTGGG	Na^+^	Antiparallel basket	wmnm	3×(d + pd)	VI	-++	saa-ssa-ssa-saa	2aa·4sa·2ss	[20]
201D	GGGGTTTTGGGGTTTTGGGGTTTTGGGG	Na^+^	Antiparallel basket	wmnm	4×(−ld + l)	VI	-+-+	(sasa)4	4as·8sa	[41]
2M6W	GGGGTTGGGGTTTTGGGGAAGGGG	Na^+^	Antiparallel basket	wmnm	4×(−ld + l)	VI	-+-+	(sasa)4	4as·8sa	[20]
5J6U	GGGGTTTGGGGTTTTGGGGAAGGGG	Na^+^	Antiparallel basket	wmnm	4×(−ld + l)	VI	-+-+	(sasa)4	4as·8sa	[20]
2MFU	TGGGTTTGGGTTGGGTTTGGG	Na^+^	Hybrid	wnmm	2×−(llp)	III	-+	(sa)4	4sa	[40]
6AC7	TGGGGTCCGAGGCGGGGCTTGGG	K^+^	Hybrid	wnmm	3×−(llp)	III	-++	saa-ssa-saa-saa	3aa·4sa·1ss	[42]
7ALU	AGGGAGGTGTGGCCTGGGCGGG	K^+^	Hybrid	wnmm	3×−(llp)	III	-++	saa-ssa-saa-saa	3aa·4sa·1ss	[43]
2JSL	TAGGGTTAGGGTTAGGGTTAGGGTT	K^+^	Hybrid	wnmm	3×−(llp)	III	-++	saa-ssa-saa-saa	3aa·4sa·1ss	[44]
186D	TTGGGGTTGGGGTTGGGGTTGGGG	Na^+^	Hybrid	wnmm	3×−(llp)	III	-++	saa-ssa-saa-saa	3aa·4sa·1ss	[45]
2GKU	TTGGGTTAGGGTTAGGGTTAGGGA	K^+^	Hybrid	mwnm	3×−(pll)	V	-++	saa-saa-ssa-saa	3aa·4sa·1ss	[46]
2O3M	AGGGAGGGCGCTGGGAGGAGGG	K^+^	Parallel	mmmm	3×(−p−p + p + p)	VIII	+++	(aaa)4	8aa	[47]
1XAV	TGAGGGTGGGTAGGGTGGGTAA	K^+^	Parallel	mmmm	3×−(ppp)	VIII	+++	(aaa)4	8aa	[48]
2LPW	AAGGGTGGGTGTAAGTGTGGGTGGGT	K^+^	Parallel	mmmm	3×−(ppp)	VIII	+++	(aaa)4	8aa	[49]
2KYP	CGGGCGGGCGCTAGGGAGGGT	K^+^	Parallel	mmmm	3×−(ppp)	VIII	+++	(aaa)4	8aa	[50]
2LEE	TAGGGCGGGAGGGAGGGAA	K^+^	Parallel	mmmm	3×−(ppp)	VIII	+++	(aaa)4	8aa	[51]
2LXQ	TAGGGTGGGTTGGGTGGGGAAT	K^+^	Parallel	mmmm	3×−(ppp)	VIII	+++	(aaa)4	8aa	[52]
2M27	CGGGGCGGGCCTTGGGCGGGGT	K^+^	Parallel	mmmm	3×−(ppp)	VIII	+++	(aaa)4	8aa	[53]
DT2-L2T4	TTGGGTGGGTTTTGGGTGGGTT	Na^+^	Parallel	mmmm	3×−(ppp)	VIII	+++	(aaa)4	8aa	[54]
2xBlock2_T258-A	GAGGAGGAGGTGTTGTGGTGGTGGTG	K^+^	Z-Parallel	mmmm	4 × Z DNA	Z	++–	(aaaa)4	8aa·4aa^5/6r^	[55]

For a definition of these descriptors, see “Materials and Methods” section.

## Materials and methods

### G4 structural descriptors

Additionally, to the classical topology (e.g. parallel, antiparallel chair…), the oligonucleotides from the reference panel were assigned a value for each of the following descriptors as described in Fig. 1:

- Conformers: a slightly more detailed topology description, following the framework of Mouawad [25], wherein antiparallel topologies are either *chair* or *basket*, and hybrid topologies are hybrid 1, 2, 3 or 4.
- Loop progression: Considers the nature of the loop, lateral (l), diagonal (d), and propeller (p), and their rotation (+ or − for clockwise and counter-clockwise, respectively), placing the 5′-residue at the bottom right of the groove facing the reader [20]. For instance, a parallel G4 will typically be (-p-p-p), simplified to –(ppp). The 14 groups defined by Webba Da Silva *et al.* are essentially subgroups of the groove type combinations defined below.
- Groove type combinations: a framework defining eight groups numbered from I to VIII characterized by their grooves, defined as narrow (n), medium (m) or wide (w), and loop progression as defined earlier [20]. For instance, the group VI contains both (d + pd) and (−l_w_d+l_n_), corresponding to different types of antiparallel basket topologies, while chair types are in the group II (both (−l_w_−l_n_−l_w_) and (+l_w_+l_n_+l_w_)).
- Tetrad loop progression, which completes the loop progression descriptor with the number of tetrads. This descriptor intents to narrow down the conformational space to a unique stacking pattern, assuming that all possible stacking patterns are described in the groove type combination framework. This is in fact not the case of e.g. 5YEY, whose stacking pattern does not match the one expected for 3×+(lll) description (*vide infra*) [37].
- GBA, expressed as the sequence of angles from 5′ to 3′ with a for *anti*, s for *syn*. This covers all possible stacking geometries and should therefore better handle non-canonical stacks. For instance, 5YEY is described with saa-ssa-saa-ssa, whereas a canonical 3×+(lll) is sas-asa-sas-asa. Brackets indicate additional base-pair or triad guanine that are stacked on external tetrads, to better handle non-canonical structures.
- GBA-stacks, expressed as the total number of all possible guanines stacks combinations present in the core or the loops of the G4 : 5′-*syn*/*anti*’-3′ stack (sa), 5′-*anti*/*syn*-3′ stack (as), *5′-syn/syn-3′* stack (ss), and *5′-anti/anti-3′* stack (aa). For 6GZN, we also considered stacks involving loop residue: [3aa·1as]·4sa. The core of the G4 is composed of 4 sa stacks while the brackets indicate four additional aa and sa stacks from four guanines that are stacked on external tetrads. This allows to better handle non-canonical structures.
- Tetrad handedness, where each tetrad viewed from the top with the 5′-end at the bottom is a + or – for anticlockwise and clockwise tetrad H-bond donor-to-acceptor orientation, respectively (Fig. 1A). Tetrad handedness is collated from bottom (5′) to top to generate the descriptor (Fig. 1B). If viewed from the bottom, all signs are inverted, e.g. 5YEY is normally described as a −++, which is equivalent to ++− when viewed from the 5′ side. Brackets indicate additional triad guanine stacking for improved non-canonical structure handling.

### Oligonucleotide synthesis

All oligonucleotides were purchased from IDT (Integrated DNA Technologies), synthesized at a 250 nmol scale with desalting. Oligonucleotides were further desalted in-house using Amicon Ultra (3 K cut-off; Merck Millipore, Cork, Ireland) in pure water.

### Calculation of Eps2Fold and IDS

All spectra were acquired with 1-cm pathlength quartz cells using a SAFAS UVmc2 double-beam spectrophotometer (Monte Carlo, Monaco) between 220 and 350 nm with the following settings: bandwidth = 2 nm, step = 1 nm, averaging time = 0.5 s. Spectra were recorded at 20°C. A detailed protocol is provided in Supplementary Information, part I.

For the acquisition of UV spectra in absence of salt, oligonucleotides were dissolved in 483.3 μl of a buffer solution composed of 10 mM Tris–HCl (pH 7.2) at strand concentrations ranging between 3.8 and 7 μM, which resulted in an absorbance spectrum with a maximum of around 1 at 260 nm. For the acquisition of the UV spectra in the presence of salt, 16.7 μl of a solution of 3 M KCl or NaCl (folded state) or LiCl (unfolded state) was added to the previous solution to yield a final salt concentration of 100 mM KCl, NaCl, or LiCl.

All spectra were blank- and baseline-subtracted, then absorbances $A$ were converted with Equation (1) to molar extinction coefficients $\varepsilon$ (M^−1^cm^−1^) across all wavelength $\lambda$ (nm), accounting for changes in analyte concentrations $C$ (M), and readily amenable to compare different cell optical pathlengths $l$ (cm).


(1) \begin{eqnarray*}
{{\varepsilon }_\lambda } = \frac{{{{A}_\lambda }}}{{l \times C}}.
\end{eqnarray*}

All UV-vis spectra are shown in Supplementary Fig. S1. Eps2Fold signatures (Supplementary Fig. S2) were calculated by subtracting the folded-state UV spectra (expressed in molar extinction coefficient) from the corresponding calculated unfolded spectra (*vide infra*). Eps2Fold spectra are concentration- and pathlength-normalized and therefore expressed in molar extinction coefficient units. Additional normalization (e.g. to [−1;1], to variance) did not yield better resolutions of similar analytes, and was therefore not used thereafter. IDS were obtained by subtracting the experimental unfolded-state spectra instead (Supplementary Fig. S3).

The calculated UV spectra were obtained following the theoretical framework described by Tataurov *et al.* [56], which relies on the nearest-neighbor model [57–60]. The calculation was implemented in R, based on our previous installment of extinction coefficients at 260 nm (${{\varepsilon }_{260}}$) determination [61, 62]. Traditionally, extinction coefficients at 260 nm (${{\varepsilon }_{260}}$) are calculated by adding ${{\varepsilon }_{i,\ i + 1}}$, the coefficients of nucleotide doublets (e.g. AA, AC, etc.), then subtracting the sum of individual nucleotide coefficients ${{\varepsilon }_i}$ (Equation 2).


(2) \begin{eqnarray*}
{{\varepsilon }_{260}} = \ \mathop \sum \limits_{i = 1}^{{{N}_b} - 1} {{\varepsilon }_{i,\ i + 1}} - \mathop \sum \limits_{i = 2}^{{{N}_b} - 1} {{\varepsilon }_i}.
\end{eqnarray*}

Here, we implemented in R the simplified Equation (3), where ${{N}_{ij}}$ represents the number of nearest-neighbor doublets, $\varepsilon _{ij}^{n - n}$ their corresponding extinction coefficients, and E denotes the sequence termini (e.g. a 3′-end A is AE).


(3) \begin{eqnarray*}
{{\varepsilon }_{260}} = \ \mathop \sum \limits_{i,\ j = A,\ C,\ G,\ T,\ E} {{N}_{ij}}\varepsilon _{ij}^{n - n}.
\end{eqnarray*}

Extinction coefficients at all other wavelengths ${{\varepsilon }_\lambda }$, necessary to produce entire spectra, are then calculated by scaling ${{\varepsilon }_{260}}$ using the parameters of the different nucleotide doublets $R_{ij}^{n - n}$, proportionally to their fraction in the ${{N}_b}$-base long sequence (Equation 4).


(4) \begin{eqnarray*}
{{\varepsilon }_\lambda } = {{\varepsilon }_\lambda }\mathop \sum \limits_{i,\ j = A,\ C,\ G,\ T,\ E} \frac{{{{N}_{ij}}}}{{{{N}_b} + 1}}R_{ij}^{n - n}.
\end{eqnarray*}

All $\varepsilon _{ij}^{n - n}$ and $R_{ij}^{n - n}$ are available on the Zenodo and GitHub repositories (https://github.com/EricLarG4/Eps2Fold) [63].

### Circular dichroism spectroscopy

CD spectra were measured between 220 and 350 nm (0.5 nm data pitch) using a Jasco J-1500 spectropolarimeter and 1-cm pathlength quartz cells. Individual spectra correspond to the average of three scans recorded at a speed of 50 nm min^−1^ with a bandwidth of 2 nm and a data integration time of 1 s. Oligonucleotides were dissolved in 500 μl of a buffer solution composed of 10 mM Tris–HCl at pH 7.2 and KCl or NaCl 100 mM at strand concentrations ranging between 3.8 and 7 μM.

The effective concentrations of oligonucleotides $C$ (M) was determined by their absorbance at 260 nm in samples devoid of potassium or sodium (i.e. in their non-folded state). CD spectra were blank and baseline subtracted, then ellipticities $\theta$ were converted to molar ellipticities $\Delta \varepsilon$ (M^−1^cm^−1^) with Equation 5 (Supplementary Fig. S4) [61]:


(5) \begin{eqnarray*}
\Delta \varepsilon = \frac{\theta }{{32980 \times l \times C}}.
\end{eqnarray*}

### NMR spectroscopy

NMR experiments were performed on a 700 MHz Bruker spectrometer equipped with a TXI probe. The 1D-^1^H-NMR spectra were recorded using a double pulse field gradient perfect spin echo (zgesgppe) pulse sequence to suppress the water signal. Spectra were recorded with a spectral width of 19 ppm, an acquisition time of 1.2 s, and a relaxation delay of 2 s. Sample strand concentration was between 100 and 200 μM in 5 mm tubes. For the acquisition of the spectra in absence of salt, oligonucleotides were dissolved in 483.3 μl of a buffer solution composed of 10 mM Tris–HCl at pH 7.2 and 10% D_2_O. For the acquisition of the UV spectra in the presence of salt, 16.7 μl of a solution of KCl or NaCl 3 M was added to the previous solution which resulted in a final salt concentration of 100 mM KCl or NaCl. All NMR spectra are shown in Supplementary Figs S5–S8.

### PCA analysis, conformation prediction, and twist angle calculations

UV and CD data processing and PCA calculations were carried out in R for IDS, Eps2Fold and CD data in the 220–310 nm range. PCA itself was performed with the PCA function from the FactoMineR R package [64]. A program with graphical user interface was developed in R with the Shiny framework [65, 66], allowing users to e.g. process their own reference set, perform PCA, leverage PCA results for conformation prediction, change the data normalization, and explore other principal components. Twist angles between consecutive guanines were calculated using the open-source PyMOL 3.0 Python package [67], with custom Python and R scripts. The software source code and other scripts are available at https://github.com/EricLarG4/Eps2Fold) [63].

## Results and discussion

### Preparation of a well-characterized G4 panel

**Figure 2.**
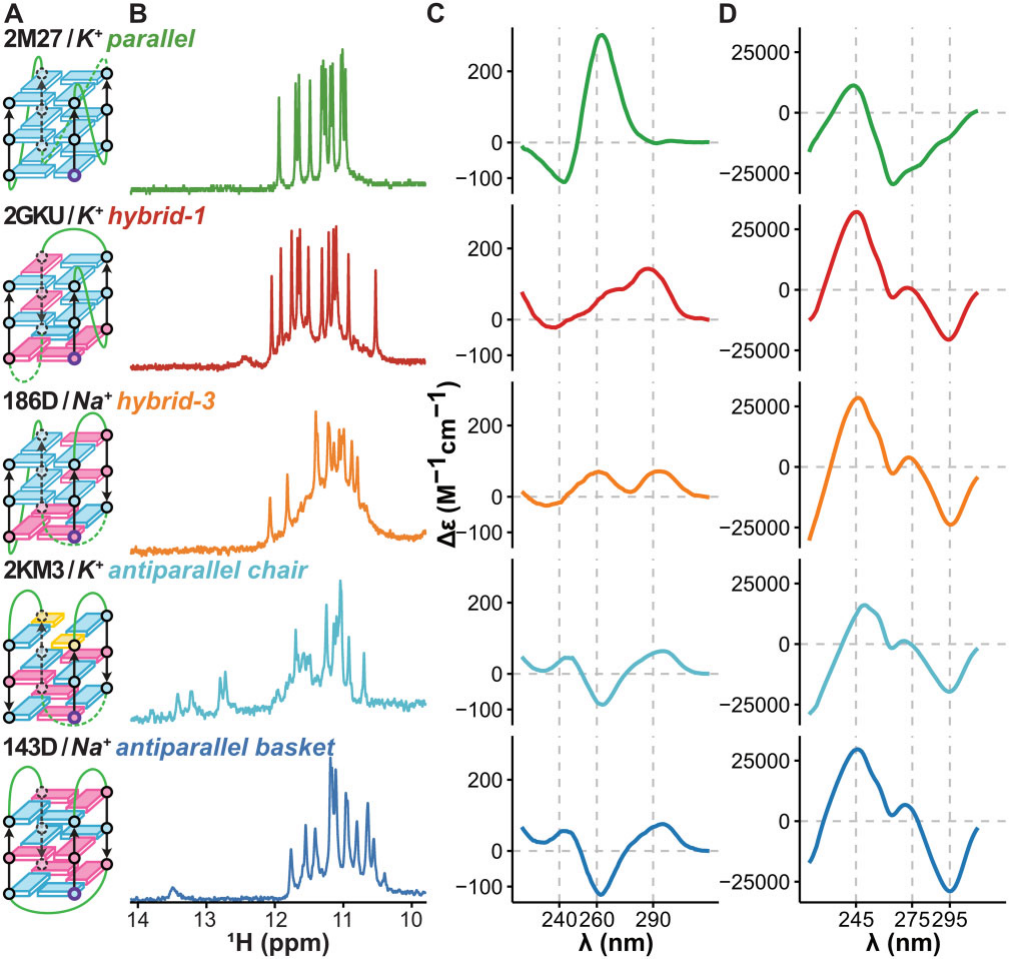
Example of spectroscopic data of the most common conformations. (**A**). Schemes of PDB deposited structures [36, 39, 45, 46, 53]. Guanines involved in tetrads are depicted as rectangles (blue: *anti* and pink: *syn* GBAs), connected by green loops. Cytosines are in yellow. (**B**). Imino proton region ^1^H-1D NMR spectra. (**C**). CD spectra and (**D**). Eps2Fold spectra are both expressed as ${\boldsymbol{\Delta \varepsilon }}$ (in M^−1^cm^1^) but are different measurements, i.e. the molar circular dichroim ${{{\boldsymbol{\varepsilon }}}_{\boldsymbol{L}}} - {{{\boldsymbol{\varepsilon }}}_{\boldsymbol{R}}}$ and the folding difference absorbance ${{{\boldsymbol{\varepsilon }}}_{{\boldsymbol{unfolded}}}} - {{{\boldsymbol{\varepsilon }}}_{{\boldsymbol{folded}}}}$, respectively.

G-quadruplex structures are known to be highly polymorphic: a given sequence can form multiple G4 conformations depending on salt concentrations, pH, mode of preparation, and type of annealing. A common and striking example of this polymorphism is that of the telomeric sequences and their variants, illustrated (non-exhaustively!) in Fig. 2A. The 22-mer human telomeric repeat in sodium (143D: A**GGG**TTA**GGG**TTA**GGG**TTA**GGG**) is in a 3-tetrad antiparallel basket conformation [39], but the 24-mer variant 2GKU (TT**GGG**TTA**GGG**TTA**GGG**TTA**GGG**A) adopts a hybrid topology in potassium [46]. Further, a mutation of the TTA loops to CTA yields an antiparallel chair, 2-tetrad conformer, capped by two GC base pairs (2KM3: AG**GG**CTAG**GG**CTAG**GG**CTAG**GG**) [36], while a mutation to TTG (186D: TT**GGG**GTTG**GGG**TTG**GGG**TT**GGG**G, *Tetrahymena* telomeric repeat) produces a different hybrid conformer [45].

**Figure 3.**
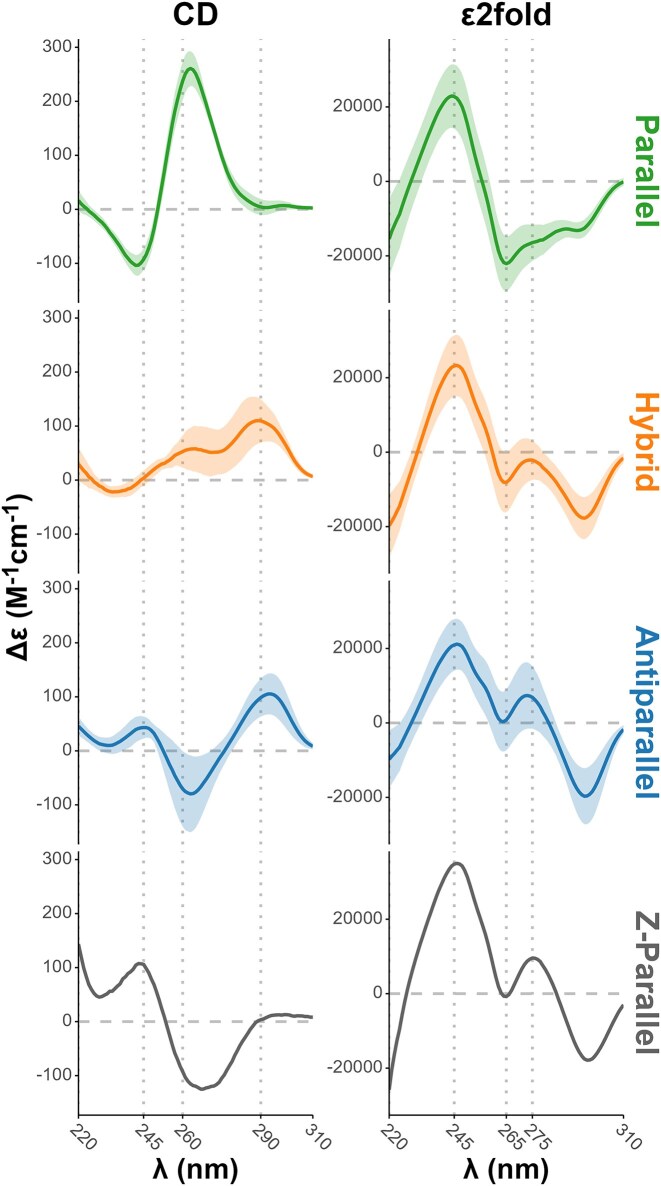
Mean CD (left) and Eps2fold (right) by topology groups (lines) ± one standard deviation (ribbon).

Thus, in our quest to evaluate if Eps2Fold signatures accurately reflect G4 conformations, we built a reference set of 30 G4 structures, well-characterized by solution-state ^1^H-NMR spectroscopy, a method of choice to accurately verify G4 folding [68]. Concretely, we verified that the published structures were reproduced by comparing the imino proton peak signatures under our conditions (Fig. 2B and Supplementary Figs S21–S24) to those reported in the literature [20, 33–55, 69]. For instance, the 12 imino proton peaks for 2M27, 2GKU, 186D, and 143D corroborate the presence of the desired three tetrads topologies (Fig. 2B). In the case of 2KM3, we detected the 8 imino proton resonances from the 2-tetrad core and the 4 imino protons of the additional C:G:C:G tetrad (Fig. 2B). We also verified that the ^1^H-NMR signatures in the absence of cations show no peaks in the imino region (Supplementary Figs S5–S8), which is consistent with the oligonucleotides being in their unfolded states, and is necessary for IDS measurements. Finally, we also verified that the CD signatures (Figs 2C and 3 and Supplementary Fig. S4) were in agreement with the G4 topologies characterized by NMR and the literature [22, 28, 29, 70]. Generally, the following CD bands are expected:

- Parallel: intense positive band around 260 nm and negative band around 240 nm;
- Z (left-handed)-Parallel: negative band around 270 nm and positive band at 240 nm;
- Antiparallel: positive bands at 295 and 240 nm and negative band at 260 nm;
- Hybrid: positive bands at 290 with shouldering at 270 nm and shallow negative band at 240 nm.

For instance, the CD spectrum of 2M27 presents a positive band at 260 nm and a negative band at 240 nm, characteristic of its parallel topology. The spectra of the antiparallel 2KM3 and 143D G4s is characterized by a positive band at 290 and 240 nm and a negative band at 260 nm. Finally, the signatures of the hybrid 2GKU and 186D G4s contain positive bands at 290 and 270 nm, and a negative band at 240 nm.

### Eps2Fold spectra present G4-dependent signatures

After validation of the G4 data set, we recorded the UV absorbance spectra of each G4 in K^+^ or Na^+^ solutions (i.e. in folded state; Supplementary Fig. S1). Then we calculated the corresponding Eps2Fold spectra by subtracting it from the unfolded-state UV spectra calculated by the nearest-neighbor method (Fig. 2D and Supplementary Fig. S2) [56, 61]. This approach allows us to rapidly generate IDS-like signatures from a single measurement, without the issues linked to G4 desalting (*vide supra*). For reference, we also recorded experimental unfolded-state UV spectra in solutions devoid of K^+^ or Na^+^ to then calculate the IDS (Supplementary Fig. S1 and Supplementary Fig. S3). As expected, obtaining truly K^+^ or Na^+^-free spectra proved to be challenging in some cases, as these cations may remain coordinated by G4s even after desalting steps, in particular for more stable species. In such cases, cation-less spectra do not faithfully reflect the unfolded state (Supplementary Fig. S1), which leads to inaccurate IDS signatures [31]. The Eps2Fold approach circumvents this issue while also eliminating time-consuming desalting steps.

In Fig. 2C, the Eps2Fold spectra display distinct spectral signatures between parallel and hybrid or antiparallel topologies; however, distinguishing between the latter two proves more challenging. This is confirmed for the whole panel by the differences in mean signatures of the G4s grouped by topologies (Fig. 3): parallel G4s are clearly distinct from non-parallel (hybrid or antiparallel) ones, but the difference between hybrid and antiparallel is not as striking as with CD (Fig. 3). Means for other groups are provided in Supplementary information (Supplementary Figs S9–S15). The Eps2Fold signature of all G4 topologies have in common a positive band at 245 nm and a negative one at 295 nm. Parallel G4s have a specific negative band around 265 nm, while this is close to zero for other topologies. This band is most prominent for the antiparallel conformations but also clearly visible in the Z-G4. These observations suggest that structural information is indeed encoded in Eps2Fold signatures, which reflect the change in molar extinction coefficients (ϵ) of the sequence between folded and unfolded states in a conformation-dependent manner, confirming circumstantial evidence gathered so far for IDS [13, 16].

A single UV spectrum is therefore sufficient not only to confirm G4 folding but also to tentatively assign topologies, similar to CD spectroscopy. For both techniques, however, more subtle differences seem to exist between conformers (Supplementary Fig. S10). For instance, we noted that the basket (143D) and chair (2KM3) type antiparallel conformers have slightly different signatures (Fig. 2), in particular the depth of the negative bands at 260 nm for the CD and the depth of the 295 nm band for Eps2Fold spectra. These more subtle differences suggest that more precise structural information is encoded in these signatures, yet their analysis requires tools beyond the naked eye. To this end, we employed PCA to sort these spectral signatures.

### Sorting Eps2Fold and CD signatures using PCA

In order to go beyond the simple visual inspection of the signatures, we used a statistical PCA approach. PCA technique transforms the original variables into a new set of uncorrelated principal components that optimally reflect the variance from the data. The original data can thus be projected on a lower-dimension system retaining the largest variance. This approach is a standard technique for reducing the complexity of complex datasets, thereby magnifying the significant differences between groups of similar analytes.

PCA was conducted on Eps2Fold spectra expressed in molar extinction coefficient units, and therefore concentration- and pathlength-normalized. The first two principal components account for most of the system variance and should therefore be sufficient to visualize differences between conformers, if any (82%; Supplementary Fig. S16A). Similarly, the CD dataset can be adequately described with two components (80% explained variance; Supplementary Fig. S16B), on data expressed in molar ellipticity units (i.e. concentration- and pathlength-normalized).

**Figure 4.**
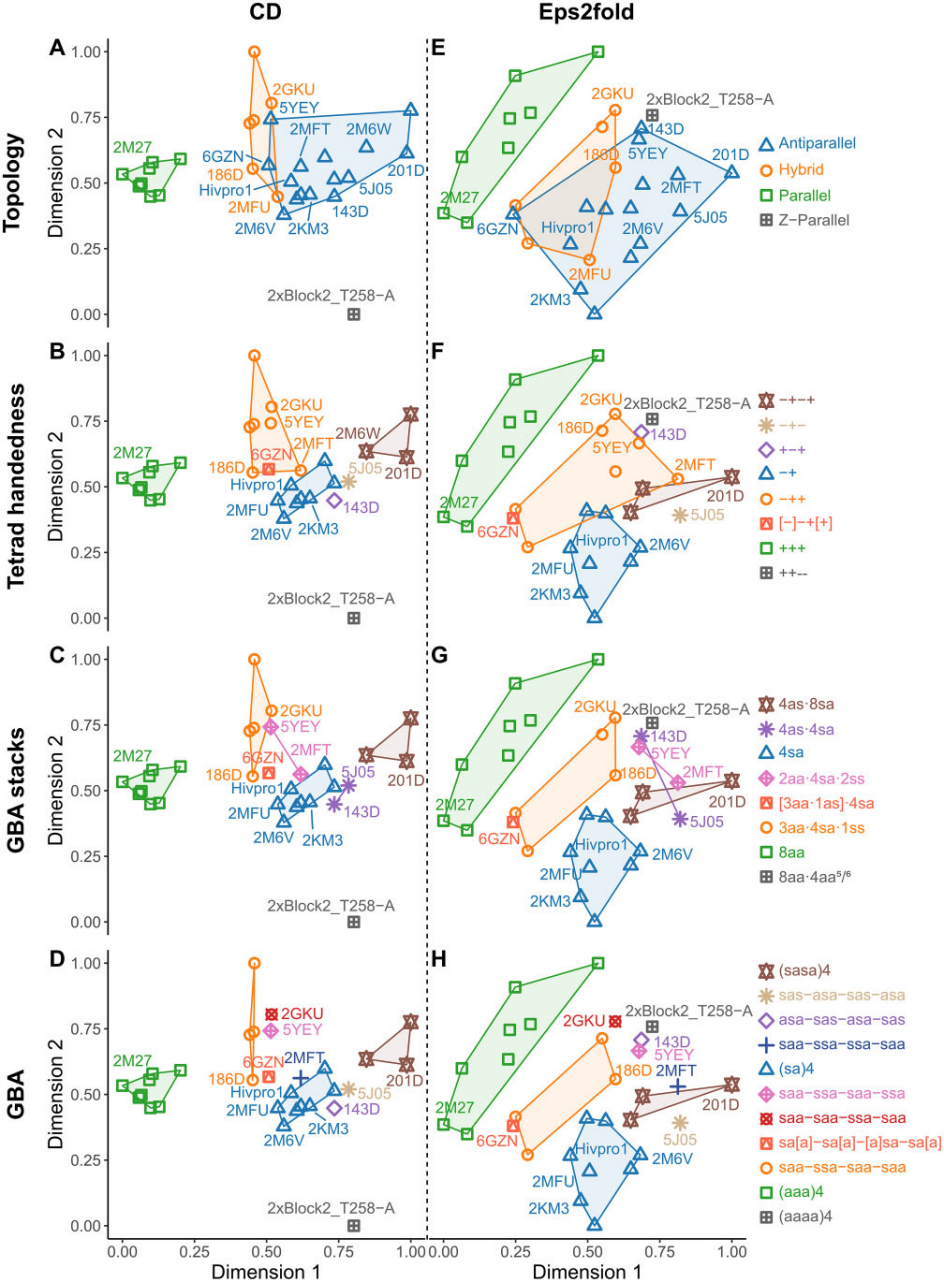
PCA results for CD (**A**–**D**) and Eps2Fold (**E**–**H**) visualized with four different structure descriptors (top to bottom). For the sake of clarity, only some oligonucleotides are labeled.

The relative contributions (direction of the eigenvectors and magnitude of their eigenvalues) of the original variables to the principal components forming the new bidimensional coordinates are given in Supplementary Fig. S17. For both CD and Eps2Fold, the main contributors are the wavelengths corresponding to the local maxima of characteristic bands, that is the well-known 240, 260, and 290 nm bands for the former and the 245, 265, 275, and 295 nm bands for the latter. In both cases, these more significant eigenvectors are well-spread on the two-dimensional plot, which allows for an efficient resolution of conformations. The PCA results using these two dimensions are shown in Fig. 4. All descriptors with full labeling are shown in Supplementary information (Supplementary Figs S18–S22). Depicting the PCA results using precise structural descriptors may more accurately reflect the key contributing elements for interpreting CD and Eps2Fold signatures. To this end, we evaluate below the resolving power and ease of use of several G4 structural descriptors, relying on either topology, strand and loop orientation, or guanine orientation.

### Evaluation of G4 structural descriptors for Eps2Fold and CD spectra analysis

#### Topology descriptors

In CD, when representing the G4s according to their classical conformations (*parallel*, *antiparallel*, or *hybrid* topologies, Fig. 4A, Supplementary Fig. S14), parallel G4s are clustered on the left (positive 260 nm and negative 240 nm bands), whereas antiparallel folds are located toward the right-hand side of the plot (positive 290 nm and negative 260 nm bands). Hybrid conformers, which are also characterized by a positive shouldering at 270 nm, are expectedly placed in between. The eigenvectors for the 270 nm shoulder characteristic of the hybrid topology are poorly resolved from the 260 nm band of parallel species (reflecting their poor resolution in CD spectra). Yet, parallel and hybrid topologies are very well resolved anyway given the difference in intensities in this region and the absence of 290 nm band for the former. For Eps2Fold, the band at 275 nm is the key contributor and explains the spread of parallel, hybrid, and antiparallel clusters from top-left to bottom-right hand corners (Fig. 4E). Although the direction of the 295 nm eigenvectors is very well resolved from those of the 245 and 275 nm bands, its contribution to the variance is small. This band is often used to infer the G4 folding but does not allow to efficiently discriminate between G4 topologies. The Z-G4 2xBlock2_T258-A included in the panel is very well resolved by CD, which was expected given its very specific CD signature (broad negative band at 260 nm and no band at 290 nm), but this is not the case by Eps2Fold. Unsurprisingly, the latter approach does not reflect changes in enantiomery contrary to CD, although it should be noted that this specific parallel Z-G4 is well separated from right-handed parallel conformers. Interestingly, both CD and Eps2Fold approaches allow for similar discrimination of parallel, antiparallel and hybrid conformers, with CD producing tighter and better resolved structural clusters. However, in both cases hybrid and antiparallel species partially overlap with the same structures being involved in the overlaps (e.g. 5YEY, 6GZN, 2MFU). For instance, the strands of 5YEY are antiparallel and classically linked by three lateral loops [37], yet its CD signature resembles that of the hybrid fold 2GKU. Interestingly, Eps2Fold signatures follow the same trend with 5YEY and 2GKU also having similar signatures. Visualization of the CD (Fig. 4A and Supplementary Fig. S18A) and Eps2Fold (Fig. 4E and Supplementary Fig. S18B) PCA results through the lens of classical *parallel*, *antiparallel*, or *hybrid* topologies shows similar degree of separation but is not precise enough. In fact, although CD is frequently employed to determine the topology of G4s based on their relative strand orientations, it is important to note that the CD signal itself arises from the guanine stacking geometry rather than the strand orientations [28, 71, 72]. The former is often linked to the latter, but not always, and this discrepancy can lead to misinterpretations of CD spectra.

#### Groove type combination and loop-progression descriptors

The groove-type combinations is related to the previous topology descriptor but divide it into finer categories (Supplementary Fig. S19). Specifically, our panel includes antiparallel chair (II) and basket (VI) conformers and hybrid 1 (V) and hybrid 3 (III) structures. However, groove-type combinations fail at improving the description of the results: antiparallel groups II and VI overlap significantly, while some overlap of these groups with hybrid folds remains. The loop progression descriptor does not significantly improve the visualization of PCA results (Supplementary Fig. S20). Using a finer sorting of data by combining the loop progression with the number of tetrads results in an even more complex picture, with smaller groups but not well resolved (Supplementary Fig. S21). Specifically, there are large overlaps between 2×−(lll), 2×(−ld+l), and 2×−(llp) in the CD results, and the situation is worse in Eps2Fold, which suffers from wider clusters. Similarly to the topology descriptor, these strand- and loop-based descriptors suffer from the same limitation as they do not directly reflect the base stackings that give rise to specific spectral signatures.

#### Glycosidic bond angle descriptors

A potential solution to this issue is to describe G4s by their guanine orientations. Every single guanine (potentially including those from additional base pairs or triads) can be accounted for by listing the glycosidic bond conformation (*syn* or *anti*) from 5′ to 3′. This GBA descriptor generates here ten different groups of right-handed G4s resulting in a completely resolved but much more complex picture with several singletons (Fig. 4D and H and Supplementary Fig. S22). For instance, 5YEY is located between groups of hybrid and antiparallel conformers, but with a singular saa-ssa-saa-ssa GBA pattern. Yet, it only differs from a cluster of hybrid-3 G4s by a single guanine from the central tetrad (186D, 2JSL, 7ALU, 6AC7: saa-ssa-saa-s**a**a). Similarly, the hybrid-1 2GKU is well resolved from the hybrid-3 cluster, while it only differs by two residues (saa-s**a**a-s**s**a-saa). This demonstrates the capacity of both CD and Eps2Fold to distinguish between structures that differ by the conformation of a single residue. Another striking feature is the excellent resolution of antiparallel G4s with four (2M6W, 5J6U and 201D: (sasa)_4_), three (2MFT, 5J05), and two tetrads ((sa)_4_). 6GZN features a two-tetrad core but is very well resolved from other (sa)_4_ G4s and is located closer to the hybrid cluster. It is in fact capped on both its external tetrads by triads that contribute to its spectroscopic signatures by generating additional homo-stacking interactions making it resemble a hybrid conformer (sa[a]-sa[a-a]sa-sa[a]).

#### Tetrad handedness descriptors

While GBA effectively identifies discrete differences between G4 structures, it is not very user-friendly and generates many singletons, which limits its predictive use. Therefore, we explored methods to retain the conveyed information in a simplified form. One way to do so is to consider entire tetrad handedness, defined by the H-bond donors-to-acceptors direction (clockwise (–) or counterclockwise (+) (Fig. 1A)). The resulting eight groups of G4s are completely resolved in CD and almost entirely by Eps2Fold (Fig. 4B and F and Supplementary Fig. S23). In the latter case, the remaining overlap can be attributed to 2MFT alone. However, this descriptor generated two singletons (5J05 and 143D), besides the single Z-G4. Determining tetrad handedness from atomic models or guanine orientations is straightforward and accounts for both guanine orientations and number of stacks (tetrads). However, this descriptor, which is seldom used in literature, may oversimplify the GBA information, resulting in overly broad clusters that obscure more detailed structural insights.

#### GBA stacks descriptors: a direct determinants of spectral signatures based on guanine-stacking geometries

**Figure 5.**
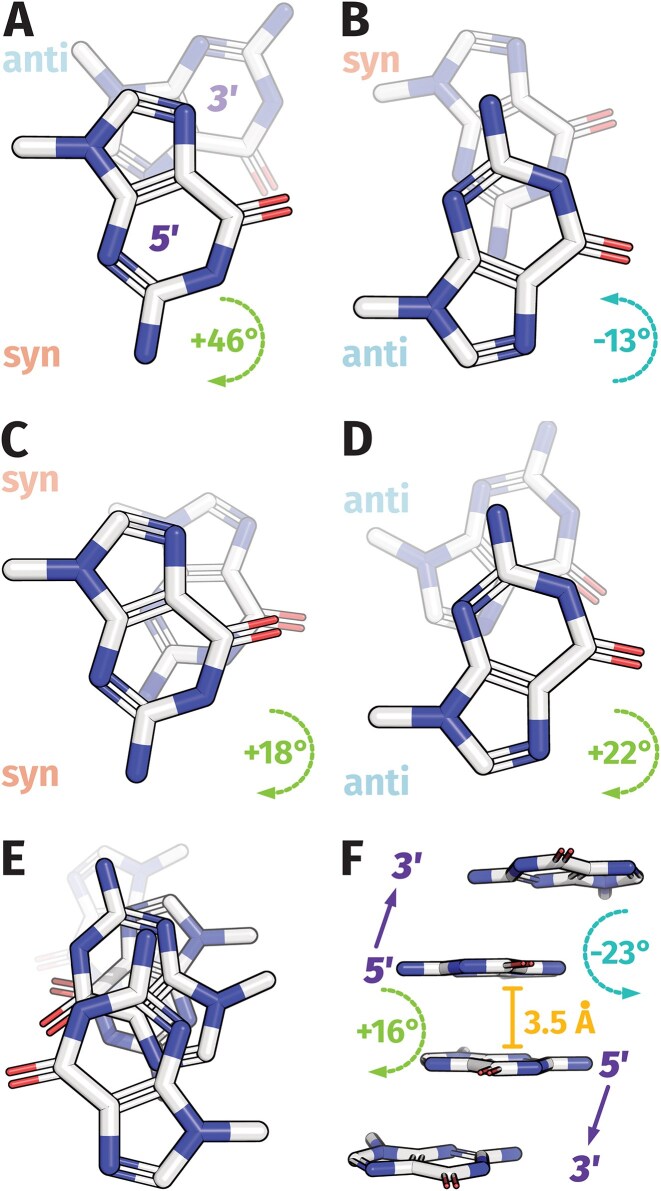
Guanine stacking overlaps differ depending on the relative glycosidic orientation: 5′-guanine/guanine-3′ stacks with (**A**) *syn*/*anti* (2GKU; G3/G4), (**B**) *anti*/*syn* (143D; G14/G15), (**C**) *syn*/*syn* (2GKU; G15/G16), (**D**) *anti*/*anti* (2GKU; G4/G5), and (**E**, **F**). left-handed *anti*/*anti*/*anti*/*anti* (6GZ6; G3/G4/G21/G20) [39, 46, 75]. The angles are the mean apparent twists across all deposited models for these example stacks, and first model geometries are shown. Mean values across the full panel are given in Table 2.

Like other nucleic acid structures, the stacking of aromatic bases is at the root of G4s formation. Annotating each GBA without accounting for guanine stacking nuances may overlook important details, as minor differences can result in distinct CD and Eps2Fold signatures. Therefore, a compromise in the form of the GBA stack representation (Fig. 4C and G and Supplementary Fig. S24) was explored. In order to interpret the spectral signatures, we first need to precisely describe these important building blocks. Depending on their GBAs (*syn* or *anti*), the stacking of two consecutive Gs from the G4 core results in a specific overlap of their 5-member or 6-member rings that defines 4 distinct stacking modes (Table 2 and Fig. 5A—D) [73]:

- 5′-*anti*/*anti*-3′ (aa): partial overlap of the 5-member and the 6-member rings (same 5/6-ring);
- 5′-*syn*/*anti*’-3′ (sa): overlap of the 5-member rings (5-ring);
- 5′-*anti*/*syn*-3′ (as): partial overlap of the 6-member rings (partial 6-ring);
- *5′-syn/syn-3′* (ss): partial 5/6-ring overlap similar to the *5′-anti/anti-3′* (same 5/6-ring).

Molecular dynamics simulations and quantum mechanics computations allowed to rank of their relative stabilities as follows: *aa* > *sa* > *as* > *ss* [73, 74].

**Table 2.** GBA patterns (*aa*, *as*, *sa*, *ss*) have the same stacking overlaps across all topologies in which they appear. Z-G4 gives rise to a very specific stacking pattern at the interface between the two 2-tetrad units

GBA-stacks^a^	Core or interface^b^	Topology	Overlap^c^	Twist angle (°)^d^	*n* ^e^
*aa*	Core	Parallel	Same 5/6-ring	22 ± 7	72
	Core	Hybrid	Same 5/6-ring	23 ± 10	16
	Core	Antiparallel	Same 5/6-ring	21 ± 2	2
	Core	Z-G4	Same 5/6-ring	-23 ± 4	8
*ss*	Core	Hybrid	Same 5/6-ring	20 ± 4	5
	Core	Antiparallel	Same 5/6-ring	19 ± 6	2
*sa*	Core	Antiparallel	5-ring	48 ± 6	60
	Core	Hybrid	5-ring	48 ± 6	26
*as*	Core	Antiparallel	Partial 6-ring	-5 ± 10	20
*aa^p6r^*	Interface	2AVJ	Partial 6-ring	10 ± 1	4
*aa^5/6r^*	Interface	Z-G4	5/6-ring	16 ± 1	4
	Interface	2LE6	5/6-ring	25 ± 1	4
*aa^6r^*	Interface	2LXV	6-ring	-0.9 ± 0.6	4
*aa^5r^*	Interface	1Y8D	5-ring	50 ± 5	4

^a^ *a* = *anti*, *s* = *syn*

^b^Stacks observed withing the G4 core or at the stacking interface between two G4 cores.

^c^Overlap geometries are shown in Fig. 5. For “same 5/6-ring” the Gs runs in the same direction, whereas they run in opposite directions for the other ones.

^d^Mean ± standard deviation across all stacks observed in the reference panel, considering all conformers when ensembles were published.

^e^Number of stacks observed in the reference panels, for each GBA/Topology group.

The dimerization interfaces between G4 units are another source of stacking interactions that are often overlooked. These interfaces can contain 5-ring and partial 6-ring stacking modes described above [73, 74]. In addition, two other stacking patterns are only observed at these dimerization interfaces. In the case of the Z-G4 [75], the hetero-stacked interface contains *anti–anti* stacks but with the two guanines running into opposite directions, resulting in a specific stacking pattern called opposite 5/6-ring overlap (***aa^5/6r^***) between the two units, not seen in the right-handed topology (Table 2). Likewise, the opposite 6-ring overlap (***aa^6r^***) has only been observed at dimerization interfaces such as the one of the dimer formed by *pilE* G4 Sequence [76]. So far, only these five different stacking overlaps present either in the G4 core or at the dimerization interfaces have been observed in the PDB. Among them, the *aa* and the *ss* stack involving a partial overlap of the 5/6-rings (same 5/6-rings) occurs from tetrads stacked with identical directionality whereas the four other overlaps arise from G-tetrads stacked with opposite hydrogen bond directionality.

As a result, visualization of the PCA according to the composition in GBA-stacks induced an identical organization for both Eps2Fold and CD signatures: Four main clusters: (i) 8aa, (ii) 4sa, (iii) 4sa·3aa·1ss, and (iv) 8sa·4as; two pairs: (i) 4sa·2aa·2ss and (ii) 4sa·4as; and three singletons: (i) 8aa·4aa* (see ZG4 below), (ii) [3aa·1as]·4sa (6GZN), and (iii) 1aa·1ss·4sa·1as (2MFU). GBA stacks retain the information about the nature and number of stacks of GBA, but in a condensed way, and allows for additional stacks to be taken into account. This representation provides a significant separation of conformations using fewer groups and singletons as compared to GBA alone or tetrad handedness descriptors.

### Key patterns and exceptions

Using the relevant G-stack descriptor (Fig. 4C and G), we outline some trends in CD and Eps2Fold signatures and highlight structural features that can influence them.

The 8aa cluster groups eight G4s that exclusively contain *5′-Anti/Anti-3′* G-stacks (same 5/6-ring) and form right-handed parallel G4s. The left-handed Z-G4 2xBlock2_T258-A is well discriminated from right-handed parallel G4s, even though both contain only *anti* guanines. In first approach, this discrimination is not surprising for CD, which is a method of choice to discriminate enantiomer-like structures. However, the CD of 2xBlock2_T258-A is not strictly mirroring that of the parallel right-handed structures. Besides, Eps2Fold is not sensitive to chirality but does discriminates this left G4 from right-handed parallel G4s. Therefore, contribution of particular GBA stack patterns of left-handed G4s impact their CD and Eps2Fold signatures. Looking more precisely to the fine structure of the left and right-handed G4s, significant differences in the G-stacking modes appear [75]. 2xBlock2_T258-A is built from two 2-tetrad cores, composed of *anti–anti* stacks (partial 5/6-ring overlaps) similar to those present in right handed G4s but with reversed twists (−23° versus +22°, respectively; Table 2) between two successive Gs (Fig. 5E) which generates the left handed helicity. However, the two 2-tetrad units are also stacked head-to-head to one another, forming a four-tetrad zigzag structure (Fig. 5F). The hetero-stacked interface contains *anti–anti* stacks with a specific stacking pattern called opposite 5/6-ring overlap, not seen in the right-handed topology (twist angle of 16°; Table 2). This generates a very specific G-stack pattern 8aa·4aa^5/6^*^r^* in comparison to the 8aa pattern of the canonical three tetrads parallel G4. These ZG4s specific stacking likely account for the particular spectra of Z-G4s and explains why they can be distinguished by an enantiomer-agnostic measurement such as Eps2Fold. In the same vein, we examined two G4 dimers displaying stacking interactions at the interface that are not observed in monomers: 2LE6 (16aa·4aa^5/6r^) and 1Y8D (16aa·2sa·2aa^5r^). 2LE6 is formed by the 5′/5′ end stacking of two identical monomers and is composed of 80% of aa stacks from the monomers and 20% of aa^5/6r^ stacks from the stacking interface. 1Y8D (93del aptamer) forms an interlocked dimer with 80% of aa stacks and 20% of sa or aa^5r^ stacks. Their CD spectra resemble those of canonical parallel monomeric G4s yet their Eps2Fold signatures are distinctly different from the 8aa cluster (Supplementary Fig. S25). Interestingly, Eps2Fold was able to detect the presence of the sa, aa^5/6r^ or aa^5r^ among a majority of canonical aa stacks while CD failed to do so.

The differences in Eps2Fold signatures observed here show that Eps2Fold spectra are sensitive to small changes in the GBA-stacking interactions. If *anti*/*anti* stacks are not equivalent between parallel and Z-G4s, this raises the question of whether such differences arise between other topologies. After analysis of our panel, we found that twist angles of a given stack are remarkably similar across all topologies in which they occur (Table 2). This further validates the use of guanine-orientation based descriptors for CD and Eps2Fold visualization.

**Figure 6.**
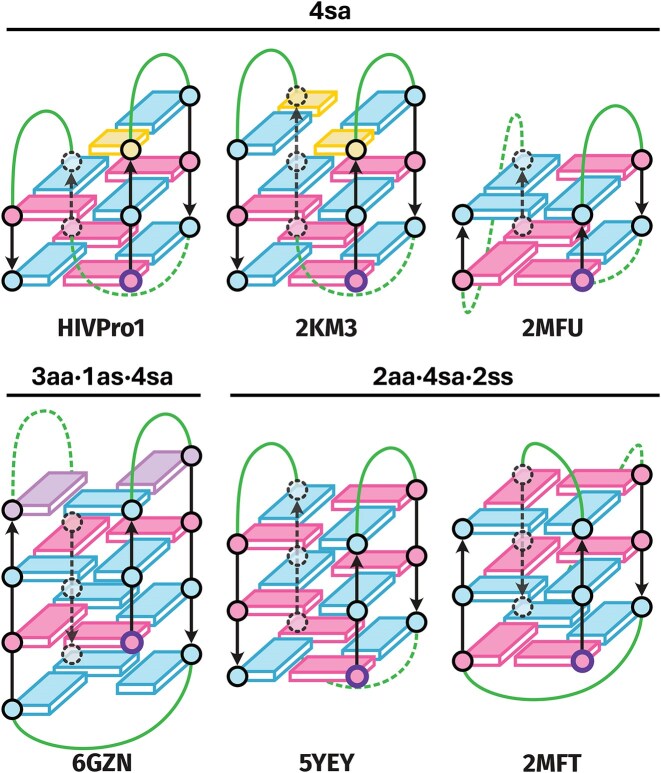
Schemes of some peculiar structures [35–38, 40, 69]. Guanines involved in tetrads are depicted as rectangles (blue: *anti* and pink: *syn* GBAs), connected by green loops. Cytosines are in yellow and adenines in purple. The 5′-end guanine is highlighted with a bold purple circle.

The 4sa cluster contains eight G4s exclusively composed of four *syn/anti* G-stacks. The presence of additional *anti*/*syn* stacks in HIVpro1 (one) and 2KM3 (two), resulting from the formation of a CG base pair and a C:G:C:G tetrad (Fig. 6), respectively, does not significantly alter their signatures. The stable *syn*/*anti* stacks of the G4-core strongly govern the CD and Eps2Fold signatures. Interestingly, the hybrid-3 2MFU contains a two tetrads core composed of four *syn/anti* G-stacks (Fig. 6), and clusters well with other 4sa G4s despite forming a very different structure. Additional stacking of Gs from the loops, similarly to HIVpro1 and 2KM3, do not significantly influence the CD and Eps2Fold signatures. This observation demonstrates once again that strand-based topology descriptors are not adequate to interpret CD and Eps2Fold signatures, which are mainly governed by the guanine stacking geometry.

The 3aa·4sa·1ss cluster is located in between the mostly antiparallel 4sa and the homogeneous, all-parallel 8aa clusters. This cluster contains five G4s presenting a mixture of four *syn/anti*, three *anti/anti* and one *syn/syn* stacks and all forming hybrid-3 type G4s except 2GKU that forms hybrid-1 type G4 (Fig. 2).

Interestingly, 7ALU and 6AC7 are offset from the cluster formed by 2JSL, 2GKU, and 186D (Fig. 4). Both feature a single-nucleotide bulge interrupting a G-tract, which affects the twists angles of some adjacent guanine stacks. In 7ALU, the bulge between G9 and G11 reduces the twist angle of the *syn/syn* stack (9° vs. a panel average of 20°; Table 2). In contrast, the following *syn*/*anti* G11/G12 stack is more twisted (57° versus an average of 48°). Nearby *anti*/*anti* stacks also show significant deviations: G3/G4 exhibits an unusually low twist (7° versus 23°), whereas G17/G18 compensates with a much larger twist (48°). In 6AC7, the adenine bulge between G9 and G11 has minimal impact on the interrupted tract: the *syn/syn* G9/G11 and *syn/anti* G11/G12 stacks maintain typical twist angles (22° and 50°, respectively). However, adjacent *syn*/*anti* stacks G3/G4 and G15/G16 display increased twist angles (59°), similar to the G11/G12 stack in 7ALU. These observations suggest that Eps2Fold may be sensitive to structural changes induced by bulges, whether at the bulge site or on neighboring stacks. Importantly, such discrimination does not seem to occur with CD (Fig. 4).

The 6GZN singleton adopts a two-tetrad antiparallel basket topology, with four *syn/anti* stacks in its core like G4s from the 4sa cluster (Fig. 6). However, both the CD and Eps2Fold signatures resemble closely those of the 3aa·4sa·1ss cluster. Indeed, 6GZN contains one extra GGG triplet stacking on the 5′-end and a AGA triplet on the other end [38], yielding a [3aa·1as]·4sa pattern that is very close to the 3aa·4sa·1ss hybrid cluster, with only one *anti/syn* stack replacing one *syn/syn* stack. Here, the addition of three stable *anti*/*anti* stacks does impact the signature of the 4 *syn/anti* stacks.

Likewise, the 3-tetrad antiparallel 5YEY and 2MFT present a G-stack pattern (2aa·4sa·2ss) close to that of the mostly hybrid 3aa·4sa·1ss cluster, with one *syn/syn* stack replacing one *anti/anti* stack (Fig. 6). As a result, both are located in between the 4sa and 3aa·4sa·1ss clusters, with 5YEY virtually colocalized with 3aa·4sa·1ss G4s. In this cluster, the shape of the Eps2Fold and CD signatures is therefore mainly governed by the number of *syn/anti* and *anti/anti* stacks coming from both the G4-core or the loops and regardless the presence of *syn/syn* or *anti*/*syn* stacks.

The 4as·8sa cluster contains G4s with four tetrads in antiparallel basket conformations. Unsurprisingly, the three-tetrad antiparallel basket 5JO5 and 143D (Fig. 2), with a closely related 4as·4sa GBA-stack pattern are localized nearby.

Overall, CD and Eps2Fold signatures are not directly governed by the relative strand orientations of G4s but rather by their compositions in stable guanine stacks arising from the G4 cores, loop residues, or dimerization interfaces. While the impact of base stacking on the CD signal has been well-documented [28, 71, 72], only very few studies deal with their impact on the absorbance spectra. Of note, TD-DFT (Time-Dependent Density Functional Theory) computations have been used to model the influence of the guanine stacking modes on the UV-absorbance spectra of G4s [77, 78]. Spectra for isolated guanine bases, non-stacked G-tetrads, and the five types of stacked G-tetrads all resulted in distinct absorption bands with the emergence of specific shoulders depending on the stacking modes. These changes are even more pronounced for the particular 5/6-ring stacking modes (aa^5/6r^) involved in G4 dimerization interfaces. This overlap between two guanines running in opposite directions exhibits heavily overlapped aromatic rings (Fig. 5E and F). These computational predictions confirm our purely empirical approach based on the comparison of experimental UV absorbance spectra recorded for thirty different G4 structures.

Overall, Eps2Fold and CD signatures yield similar discrimination power between G4 structures, although CD is somewhat more resolutive. Both allow excellent resolutions between most structural groups, especially when defined by their guanine orientations (GBA, GBA stacks, and tetrad handedness).

## Conclusion

In this work, we propose a new and simple spectroscopic method to detect G4 formation and assign topology. This approach may be useful for scientists who need to determine if a G4 is formed and potentially assign a basic topology (e.g. parallel or not), as well as for experts on G4s who seek to gain additional structural insights.

Below, we summarize the salient points of this work and give a few recommendations:

- Eps2Fold can routinely be used to detect G4 formation and infer a topology. Eps2Fold is not intended to replace CD, but rather to serve as a complementary technique. Its main advantages are its speed, simplicity, and scalability: it requires only absorbance spectra, can be performed without specialized equipment, and is likely amenable to high-throughput formats, which we aim to implement in future work.
- Eps2Fold signatures are the same as IDS or TDS but are not affected by salt contamination or the temperature dependence of absorption coefficients. Similar to CD, the UV data utilized in this study are expressed in units of M^−1^cm^−1^, allowing for the analysis of samples at any concentration within the instrument's linear range. Additionally, users have the flexibility to employ any spectrophotometrically transparent buffer and temperature conditions to either promote or inhibit folding.
- Interpretation of CD and Eps2Fold by the naked eye is not always sufficient, given that small changes may be meaningful. Approaches that highlight key differences and allow a subsequent clustering of analytes like PCA should preferentially be used. To facilitate this work, we have released a program with GUI allowing to import and filter data, set up and perform PCA, and visualize the result through the lens of different visual descriptors (source code available at https://github.com/EricLarG4/Eps2Fold and on Zenodo) [63]. We made the program available on a web server, but users that do not wish to upload their data online can run the R shiny app locally (requires R), in a Docker container (requires Docker) or using their web browser (solution relying on the shinylive framework [79]; no installation required).
- A given combination of guanine stacking geometries gives rise to specific CD and Eps2Fold signatures that can be resolved with a remarkable, and frankly unexpected, granulometry. Qualitatively, both methods follow remarkably similar structure separation trends, but CD is quantitatively more resolved. Both techniques generate small numbers by subtracting two similar larger numbers, which is not ideal in terms of accuracy and precision. This is more prominent for Eps2Fold, hence its larger spread of data. Additionally, CD signatures are inherently more different across conformers, which further increases their resolution.
- Discrete changes in guanine orientations can give rise to signatures that can be resolved. Because left-handed G4s are specific constructs differing from all right-handed topologies, they can be resolved even with a chirality-agnostic method like Eps2Fold. Eps2Fold is particularly efficient at discriminating dimers from parallel monomers, unlike CD. Similarly, Eps2Fold may also be able to distinguish G4s with bulges, whereas CD does not seem to, but the analysis of a larger panel of bulge-featuring structures will be necessary to confirm these initial findings.
- As a result, interpretation of the data requires the use of descriptors accounting for the relative orientation of guanines, whether at the individual (GBAs) or more global levels (GBA stacks, tetrad handedness). Conformer descriptions based on the orientation of strands and/or loops can often be adequate but cannot be recommended because the guanine orientations do not always follow the pattern expected of a given topology (e.g. 5YEY). At least in the context of conformation determination and description, this study highlights the limitations of classical topology descriptors.
- More precise structural descriptors generate numerous subgroups, which complicates the picture. In the context of Eps2Fold and CD data processing, the GBA-stack is a good compromise, as it reflects fairly intuitively the classical topologies but clusters them in a way that better accounts for the nature and number of stacks.
- Like all bulk methods, Eps2Fold and CD output the mean spectrum of all mixed conformers weighted by their relative abundances. When comparing the data of an unknown sample to a reference panel, the analyst must therefore be cautious in both sample preparation, which can greatly affect the conformational equilibrium, and its interpretation of the results. Orthogonal methods must be used to assess the structural purity of the sample.

Looking ahead, we aim to extend this approach to a broader range of secondary structures beyond G4s, in both DNA and RNA oligonucleotides. In particular, assessing the generalizability of Eps2Fold in terms of resolving power will be essential, and may require the development of improved structural descriptors tailored to other secondary structure motifs.

## Supplementary Material

gkaf953_Supplemental_File

## Data Availability

The software source code and other scripts are available at https://github.com/EricLarG4/Eps2Fold and https://zenodo.org/records/15980478.
