# Supplementary material for: Eps2Fold: a rapid method to characterize G-quadruplex DNA structures using single absorbance spectra
Source: Nucleic Acids Res. 2025 Sep 29;53(18):gkaf953. doi: 10.1093/nar/gkaf953 (PMC12477609; doi:10.1093/nar/gkaf953)
Supplement: gkaf953_Supplemental_File [file gkaf953_supplemental_file.docx]

E2Fold: A Rapid method to characterize G-quadruplex DNA structures using single absorbance spectra

Eric Largy^1^*, Aurore Guédin^1^, Amani Kabbara^1^, Jean-Louis Mergny^1,2^* and Samir Amrane^1^*

^1^ Univ. Bordeaux, CNRS, INSERM, ARNA, UMR 5320, U1212, F-33000 Bordeaux, France.

^2^Laboratoire d’Optique et Biosciences, Ecole Polytechnique, CNRS, INSERM, Institut Polytechnique de Paris, 91120 Palaiseau, France.

* Corresponding authors

SUPPORTING INFORMATION

## ****Pages SII-III: Protocol for Eps2Fold Analysis of Oligonucleotide Folding****

## ****Pages SIV-XXVII: Supplementary figures****

## ****Protocol for Eps2Fold Analysis of Oligonucleotide Folding****

### ****1. Oligonucleotide Preparation****

#### **1.1 Synthesis and Initial Dissolution**

- **Source**: Any oligonucleotide supplier such as **IDT**, **Eurogentec**, or **Eurofins**
- **Typical Scale**: ~40 or **200 nmol**
- **Purification**: Desalted or RP-Cartridge
- **Initial dissolution**: Dissolve the oligonucleotide in **500 µL of ultrapure water**

#### **1.2 Desalting and Concentration**

- **Device**: **Ultra-0.5 mL Ultra-centrifugal filter**, 3 kDa cut-off (Ex **Amicon**, Merck Millipore or Sartorius)
- **Procedure**:
  1. Load the 500 µL into the filter unit.
  2. Centrifuge per manufacturer’s instructions.
  3. Wash **2–3× with ultrapure water** to remove salts.
  4. **Concentrate** the eluate to **~300–500 µM**, using a **Nanodrop** to monitor A260.

### ****2. Buffer Preparation for UV absorbance****

- Prepare a **10 mM buffer** solution at the desired pH.
- The buffer should be:
  - **Spectrophotometrically transparent** in the UV range, down to 220 nm.
  - Stable at the chosen experimental temperature
  - Compatible with the oligonucleotide folding state
- Suitable buffer systems include:
  - **Tris-HCl**
  - **HEPES**
  - **Cacodylate**
  - **Phosphate**
- Add salt directly to the buffer depending on the oligonucleotide and desired topology:
  - Recommended salts: **KCl** or **NaCl**
  - Final salt concentration: **10–150 mM**
- Mix thoroughly to ensure homogeneity
- The buffer will be used for final dilution of the oligonucleotide before UV or CD acquisition

### ****3. Sample Preparation****

- Dilute the concentrated oligonucleotide stock in the **prepared buffer (500µl)** to a final concentration of **2–5 µM**. For an oligonucleotide composed of 20 nucleotides this will yield to an optimal optical density of **0.5–1.0 OD at 260 nm.**
- Transfer the solution to a **1 cm pathlength** Quartz **Cuvette**.

### ****4. UV-absorbance Spectra Acquisition****

#### **4.1 Instrumentation**

- **Device**: UV-visible spectrophotometer (*e.g.*, SAFAS Uvmc2 or UVIKON XS/XL or equivalent)
- **Cuvette**: Quartz, **1 cm pathlength**

#### **4.2 Acquisition Settings**

- Wavelength: **220–350 nm**
- Bandwidth: **2 nm**
- Step size: **1 nm**
- Averaging time: **0.5 s**

#### **4.3 Temperature Control**

- Measurement temperature: Depending on the melting temperature of the structure measurements can be performed between **5°C and 40°C**
- **Flush with dry air** at low temperatures to avoid condensation in cuvettes

#### **4.4 Spectra acquisition**

- Measure a **blank spectrum** (buffer with no oligonucleotide)
- Then measure the spectrum of the oligonucleotide and substract the blank by applying **blank correction** as necessary

### ****5. Eps2Fold and PCA Analysis****

#### **5.1 Upload Data**

- Visit: [**https://github.com/EricLarG4/Eps2Fold**](https://github.com/EricLarG4/Eps2Fold)
- Download the user data template
- **Open the file** using Excel or a plain-text editor.
- **Paste your spectra into the template** and save it with the desired name.
- **Upload the User data file**

#### **5.2 Analysis Workflow**

1. The application computes automatically the **Eps2Fold** spectra from uploaded data.
2. Performs **Principal Component Analysis (PCA)** to classify the spectra.
3. Each input oligonucleotide spectrum is **projected onto the reference panel** for structural comparison and interpretation.

## UV spectra


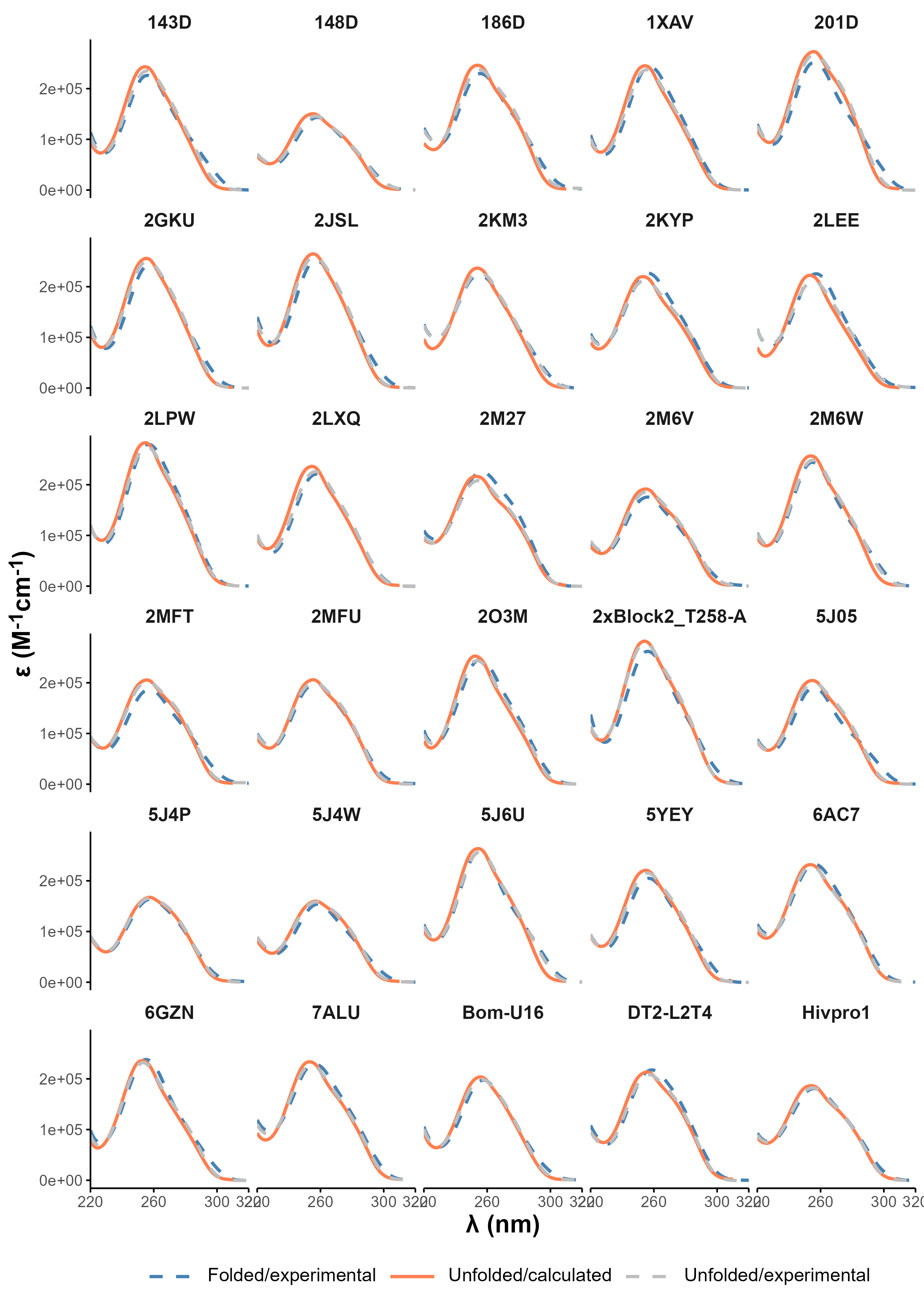


Figure S1. Experimental and calculated UV absorbance spectra of the oligonucleotides from the panel

## Eps2Fold panel


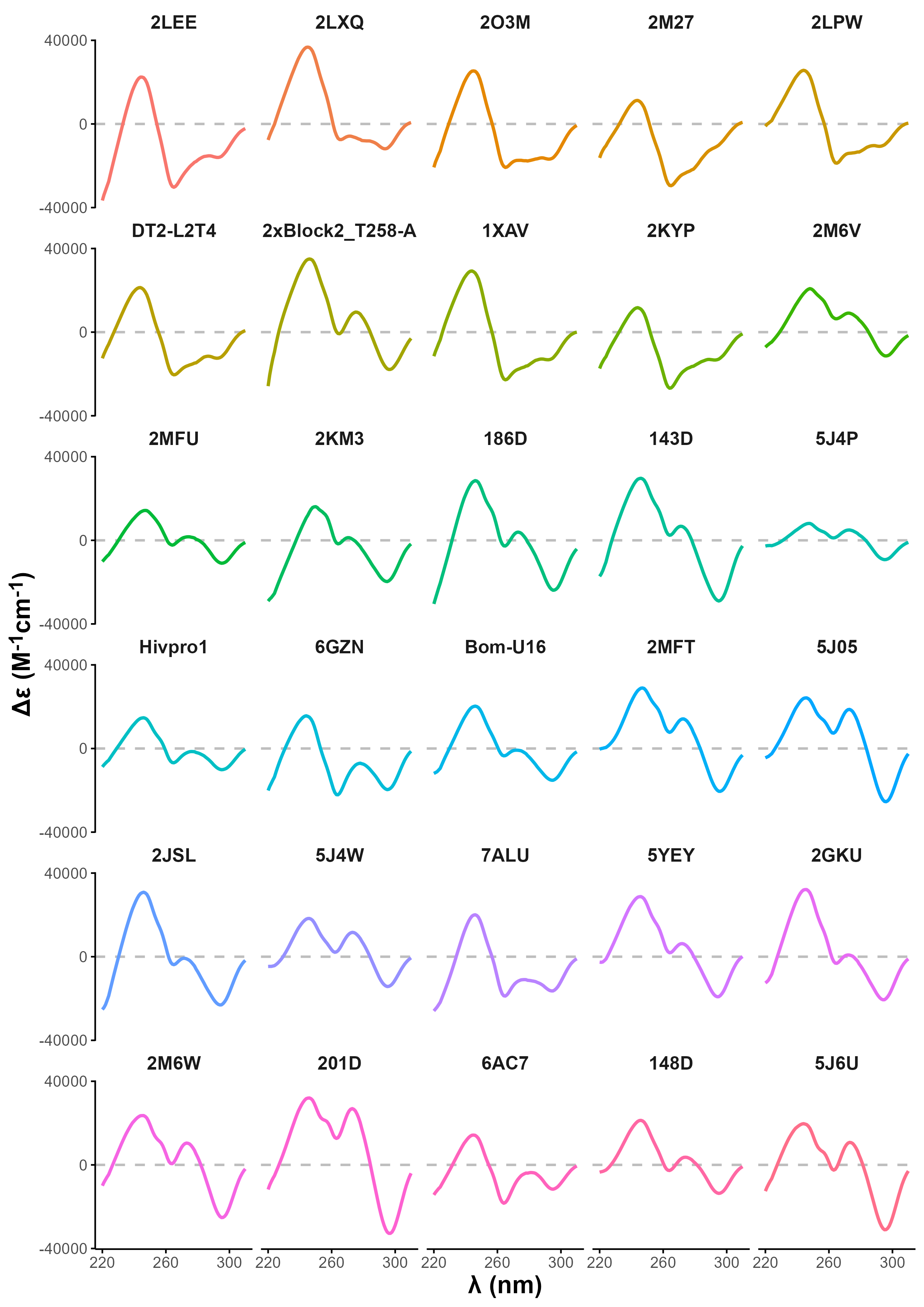


Figure S2. Eps2Fold for the oligonucleotides from the panel

## IDS panel


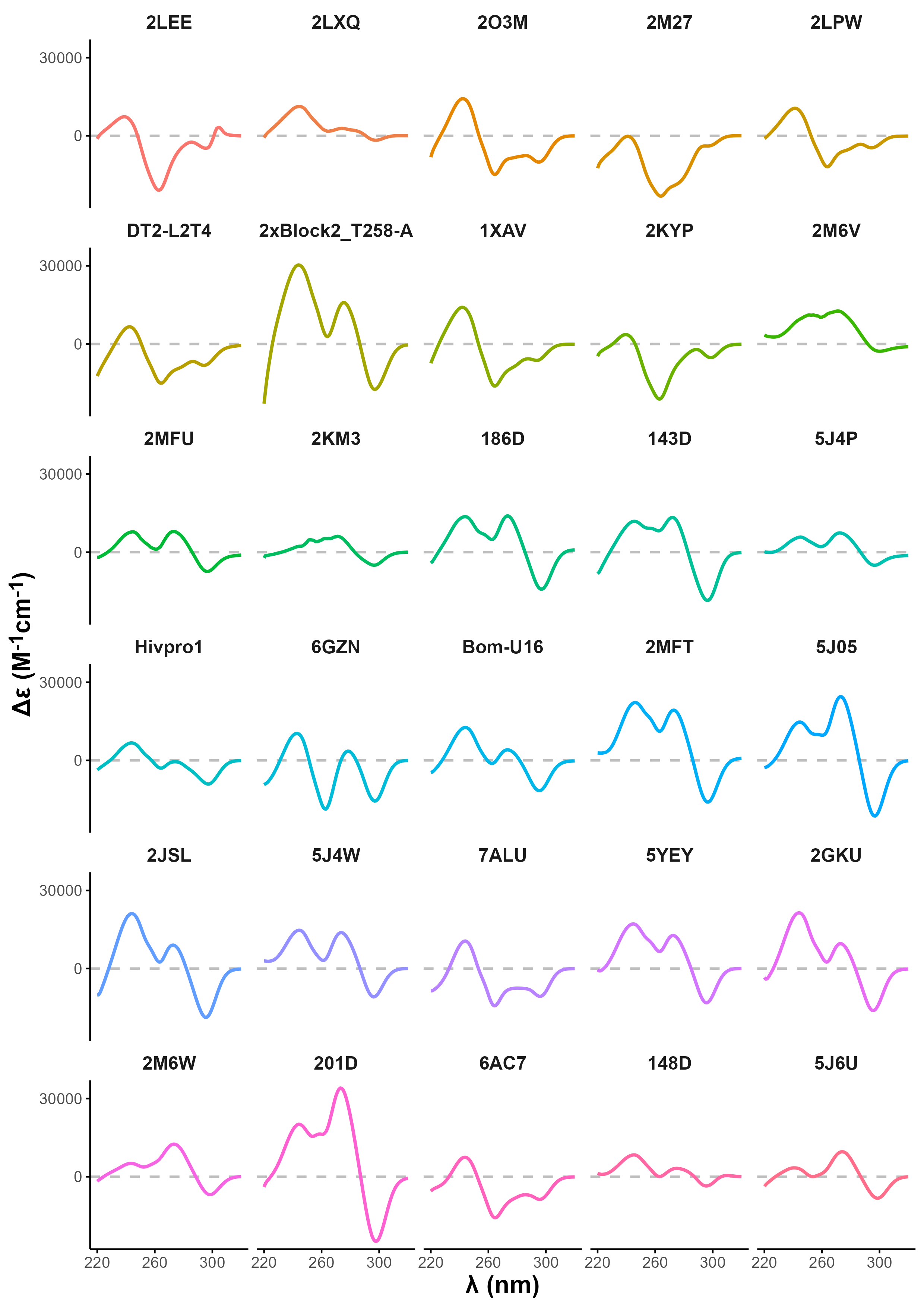


Figure S3. IDS for the oligonucleotides from the panel

## CD panel


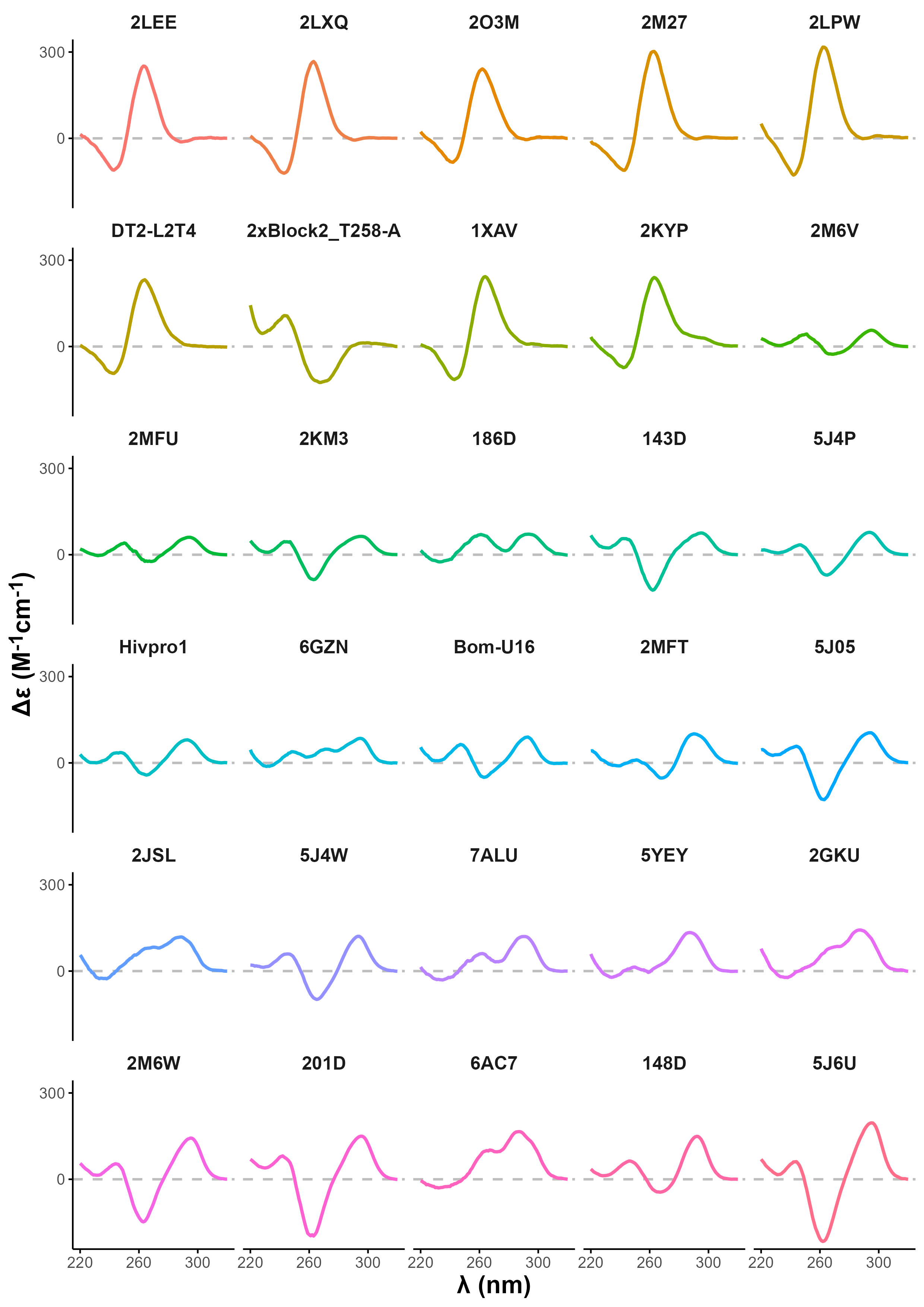


Figure S4. CD for the oligonucleotides from the panel

## NMR spectra

Figure S5. Imino proton region ^1^H-1D NMR spectra with or without salt

Figure S6. Imino proton region ^1^H-1D NMR spectra with or without salt

Figure S7. Imino proton region ^1^H-1D NMR spectra with or without salt

Figure S8. Imino proton region ^1^H-1D NMR spectra with or without salt

## Mean signatures

### Topology


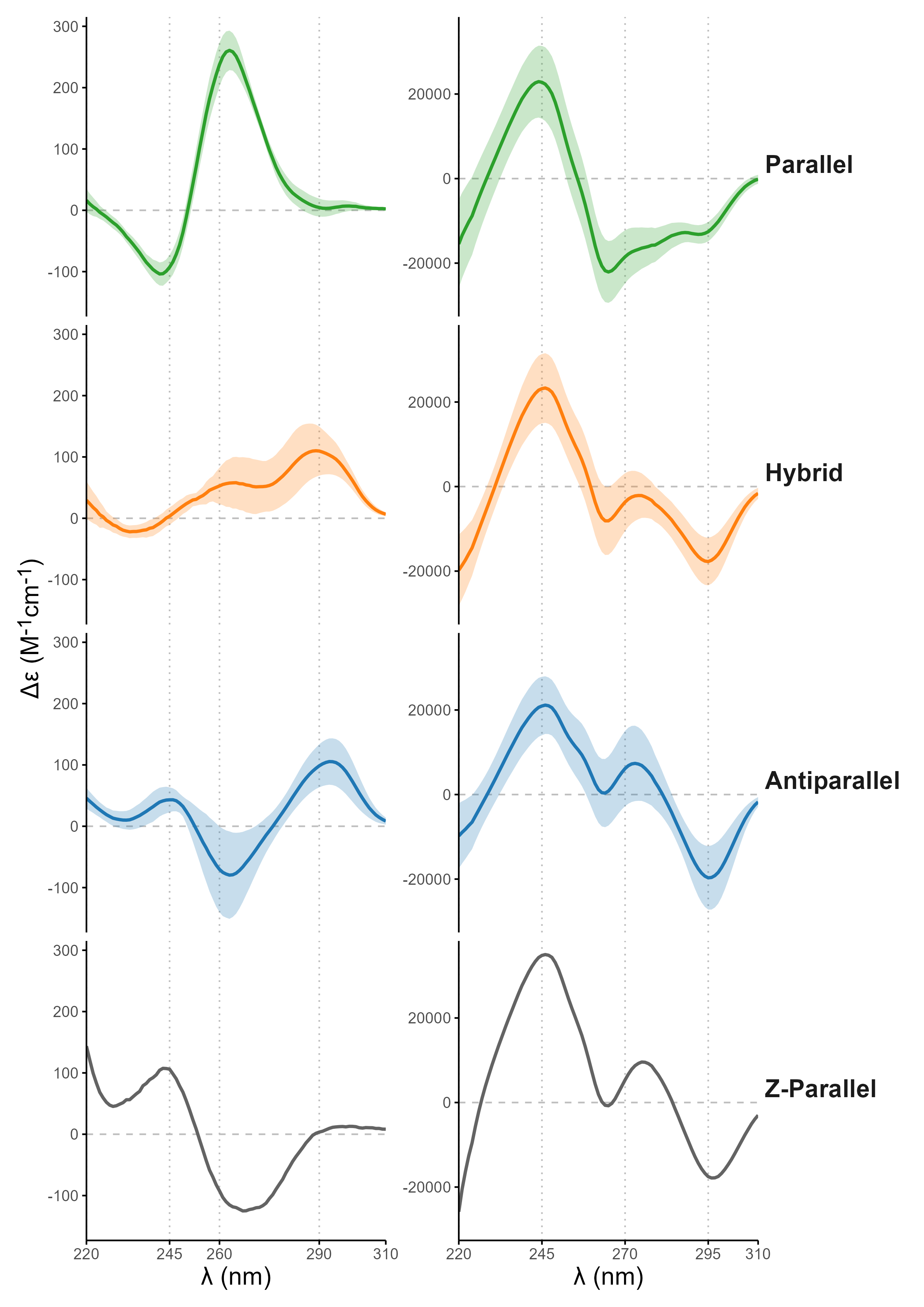


Figure S9. Mean CD (left) and ε2fold (right) signatures (lines) ± one standard deviation (ribbon) grouped by topology

### Conformers (groove type combination)


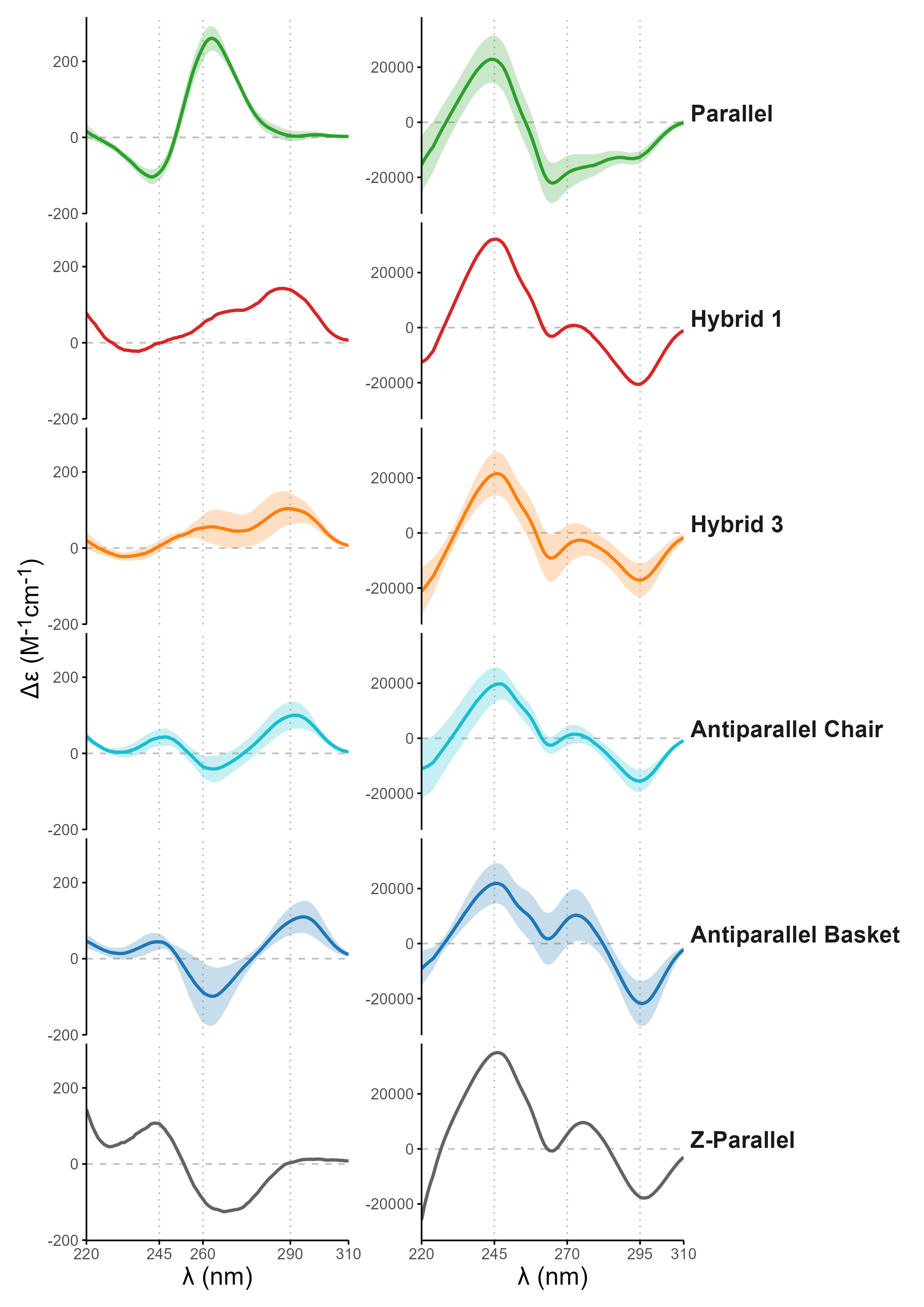


Figure S10. Mean CD (left) and ε2fold (right) signatures (lines) ± one standard deviation (ribbon) grouped by conformer (or groove type combination)

### Loop progression


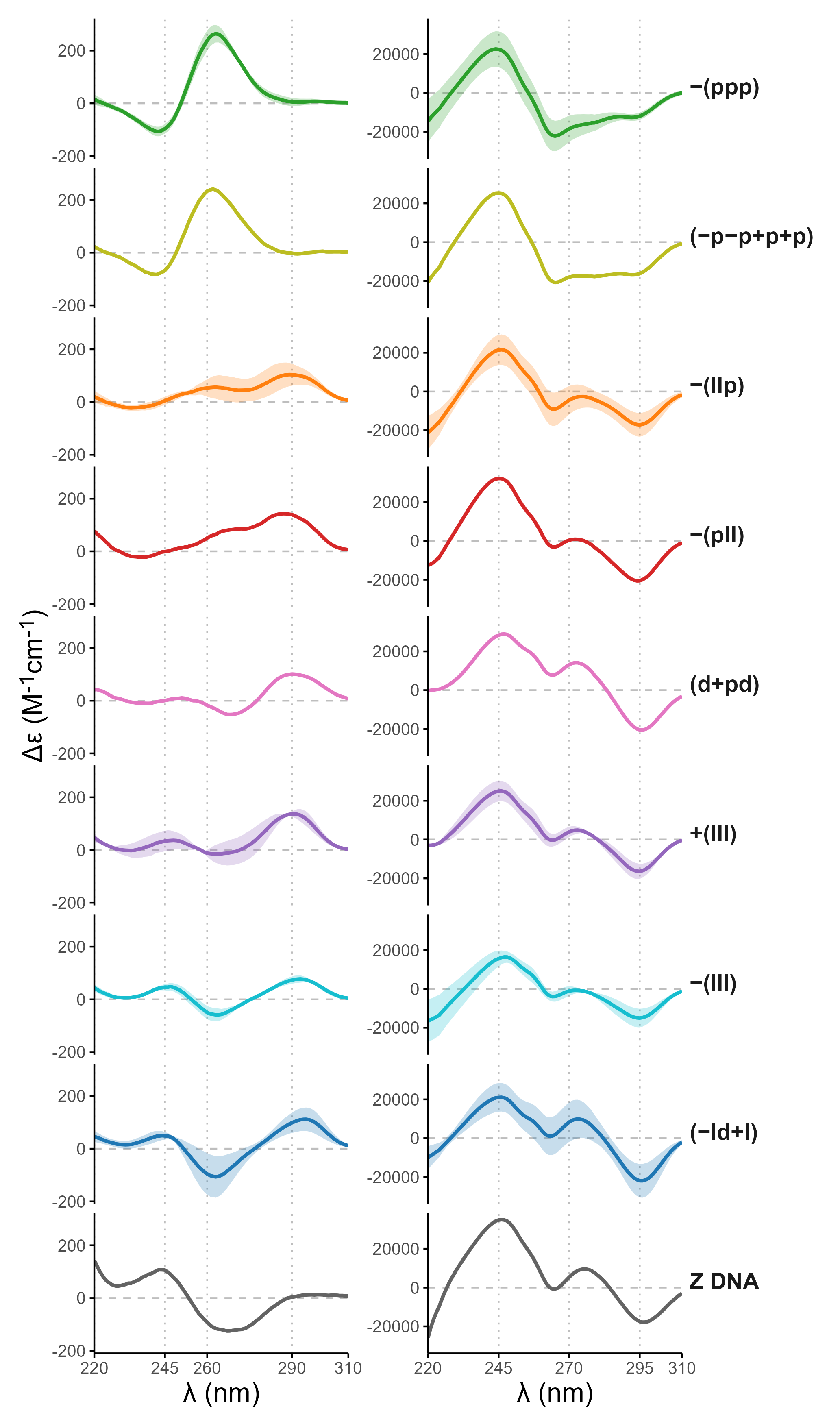


Figure S11. Mean CD (left) and ε2fold (right) signatures (lines) ± one standard deviation (ribbon) grouped by loop progression

### Tetrad x Loop progression


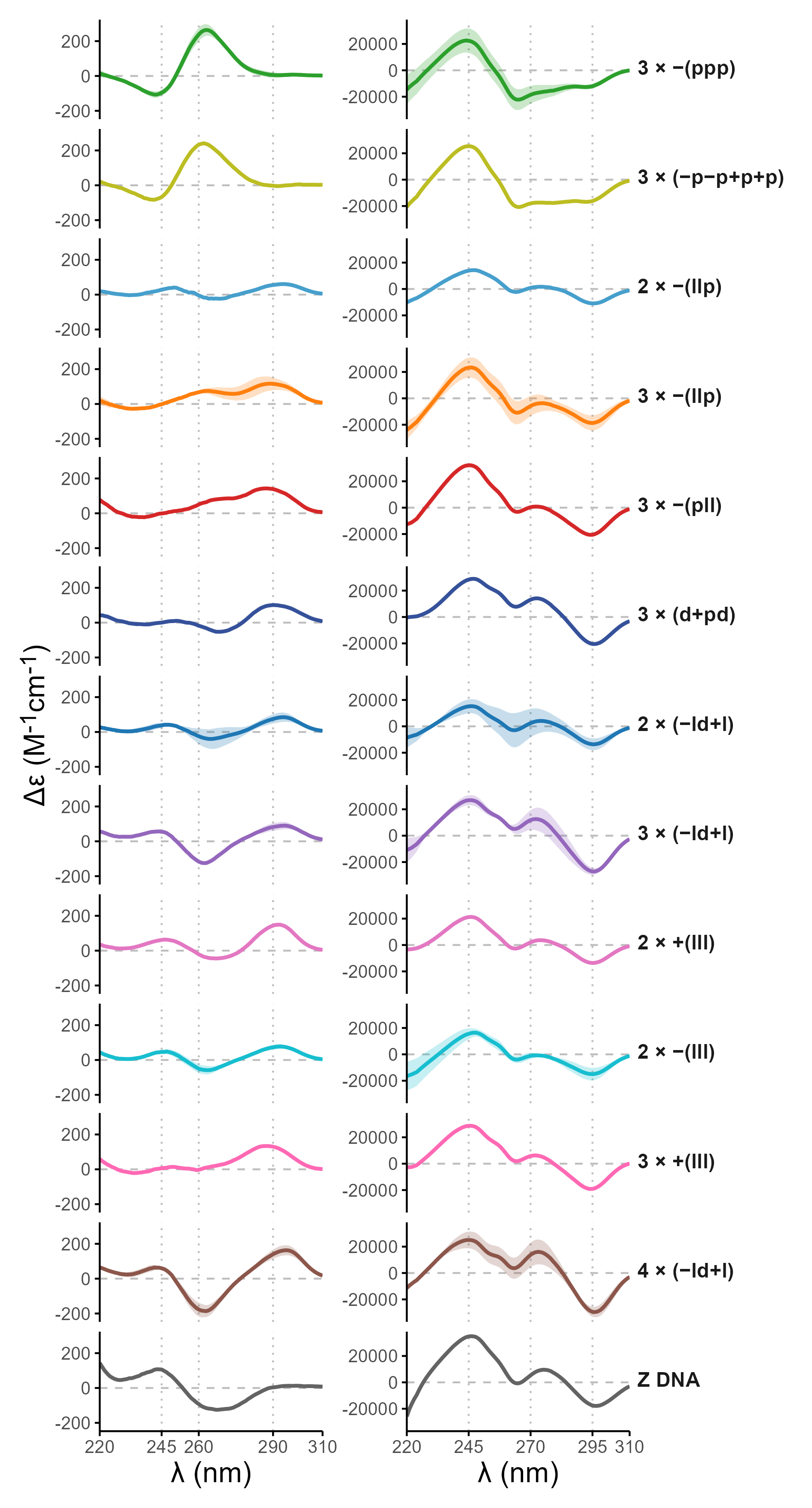


Figure S12. Mean CD (left) and ε2fold (right) signatures (lines) ± one standard deviation (ribbon) grouped by tetrad x loop progression

### Tetrad handedness


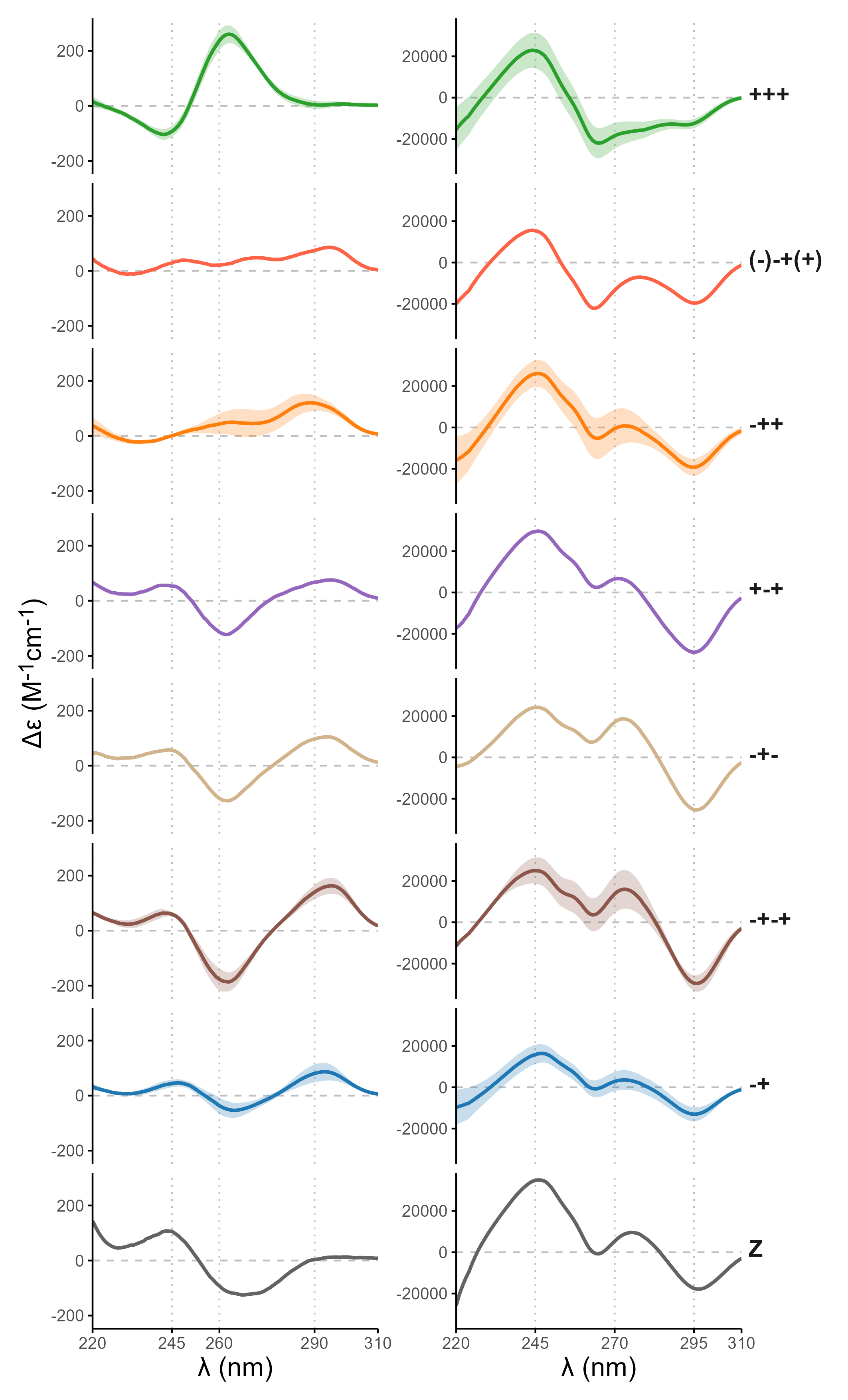


Figure S13. Mean CD (left) and ε2fold (right) signatures (lines) ± one standard deviation (ribbon) grouped by tetrad handedness, shown on the right

### GBA stacks


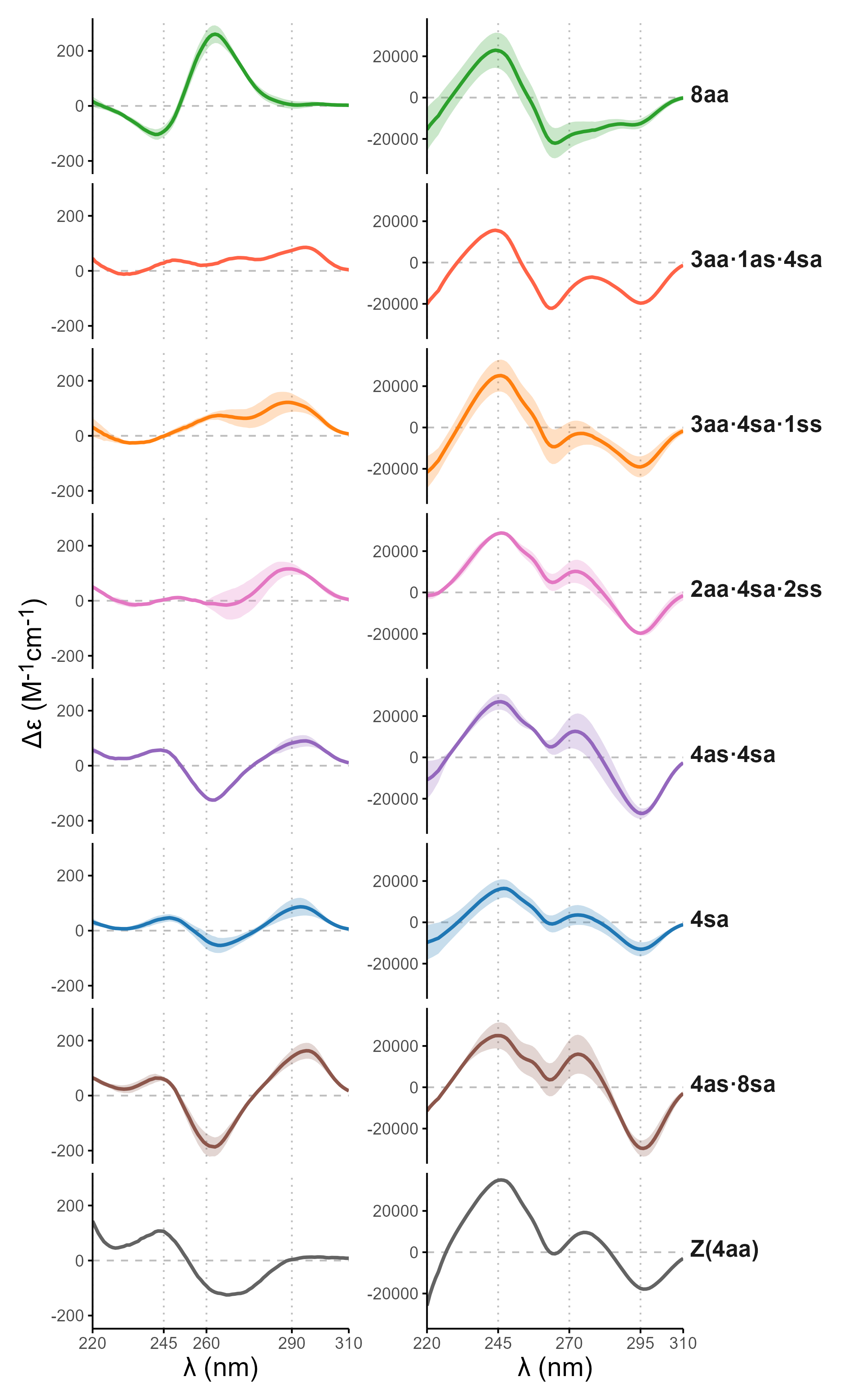


Figure S14. Mean CD (left) and ε2fold (right) signatures (lines) ± one standard deviation (ribbon) grouped by GBA stacks

### GBA


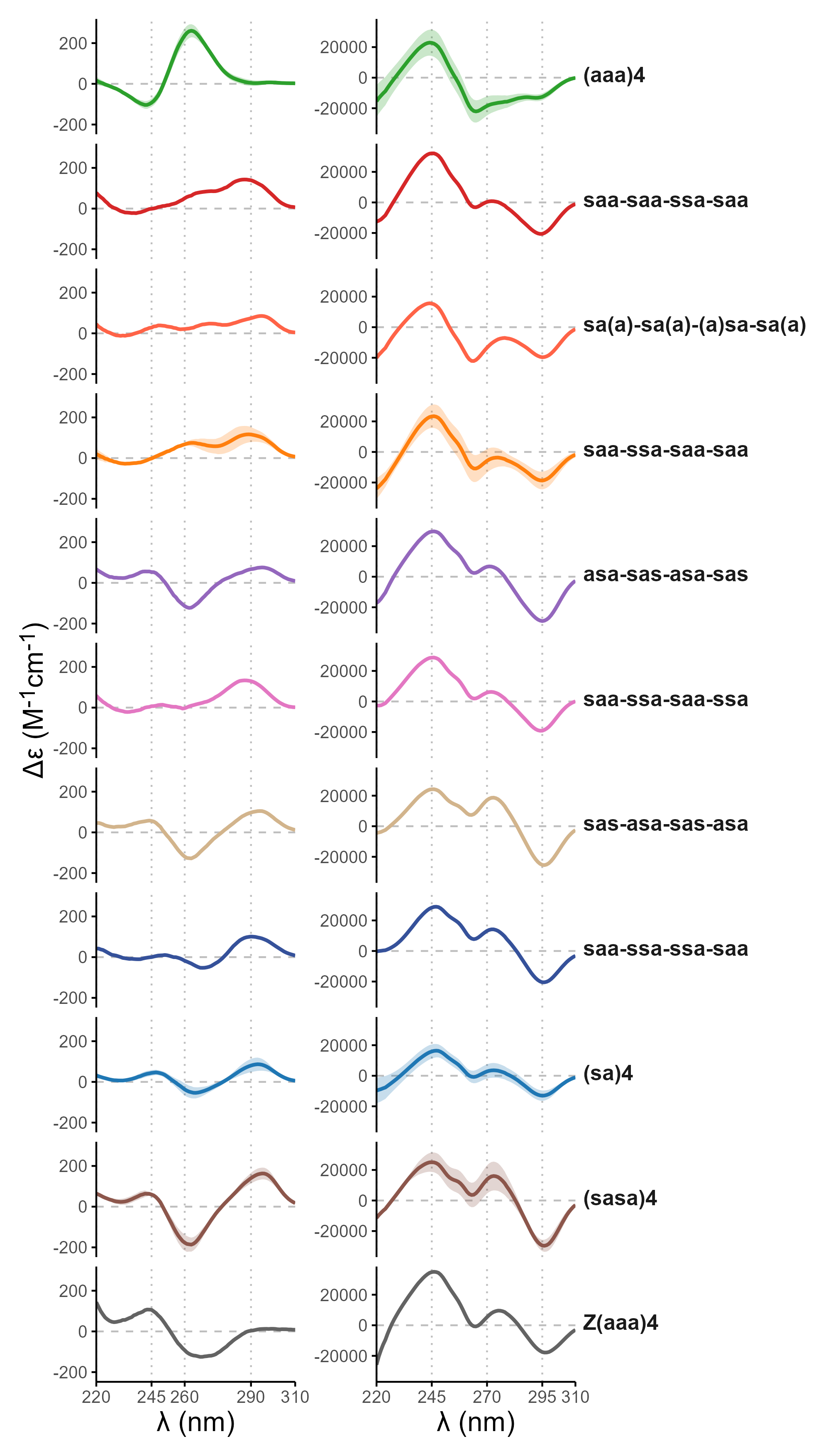


Figure S15. Mean CD (left) and ε2fold (right) signatures (lines) ± one standard deviation (ribbon) grouped by topology

## PCA diagnostics


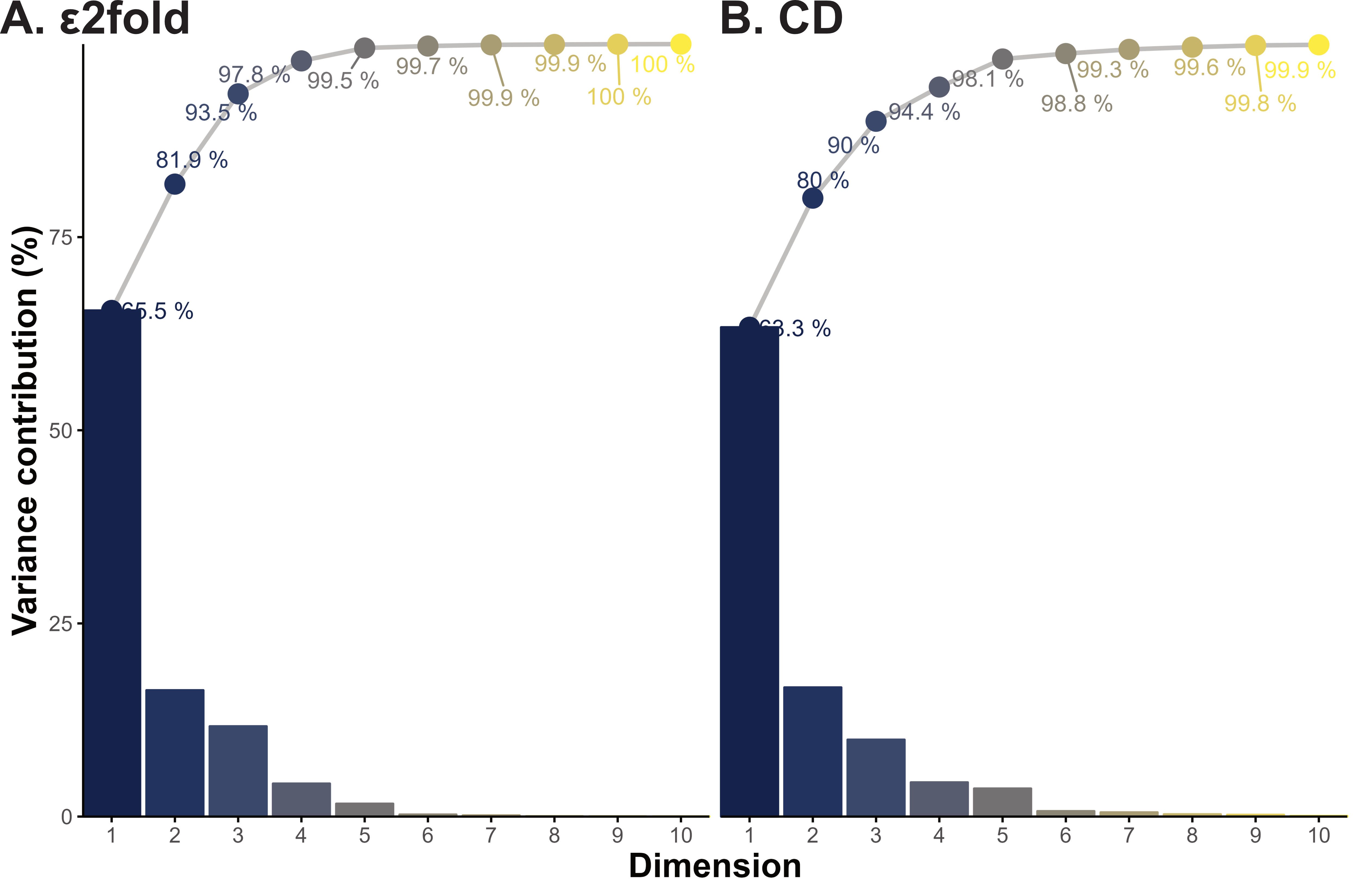


Figure S16.Contributions of the dimensions to the system variance (individual: bars, cumulative: points) for ε2fold (left) and CD (right).


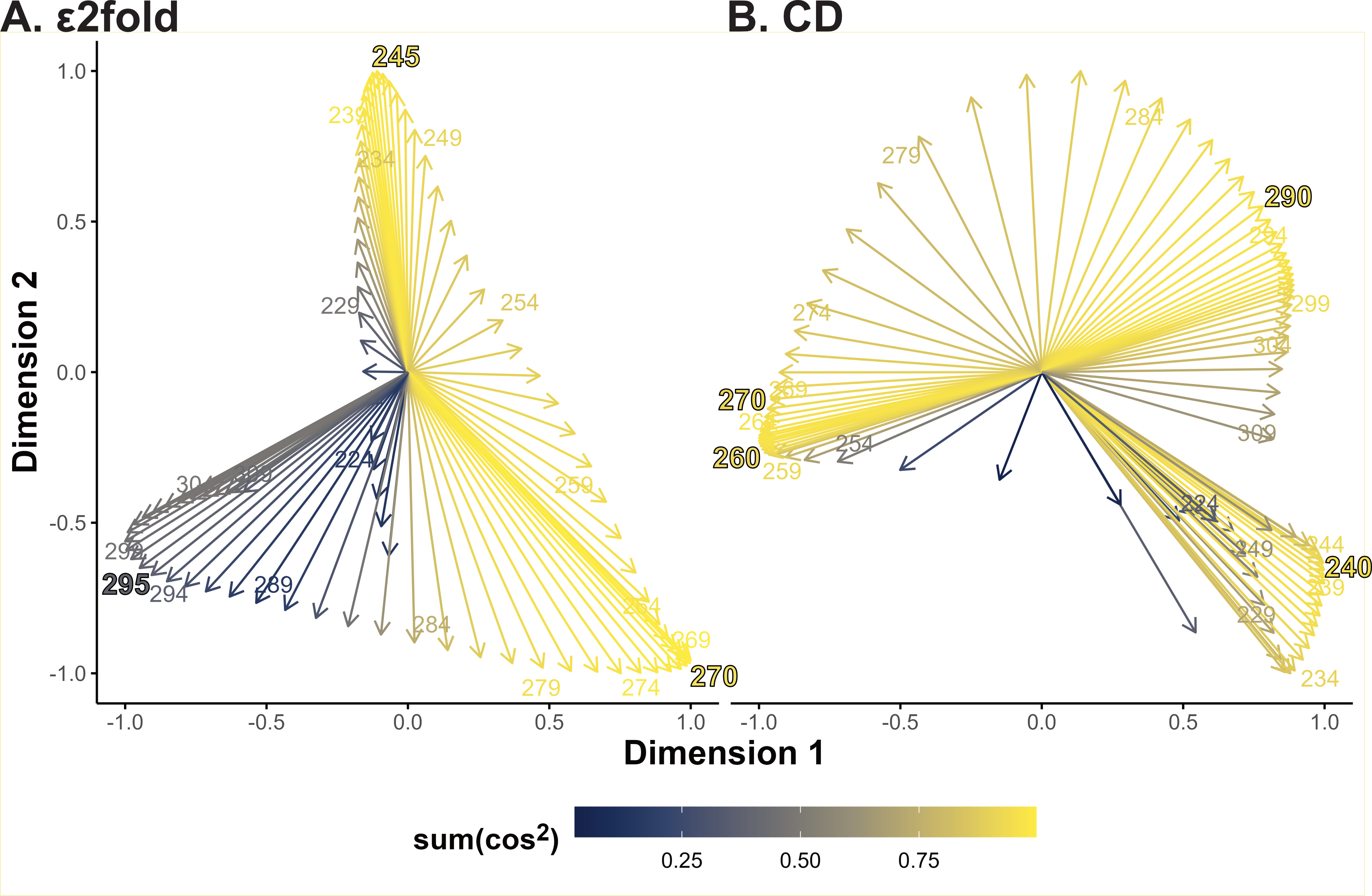


Figure S17. Variable contributions in ε2fold (left) and CD (right) PCA, colored by the sum of squared cosine.

## PCA results

### Topology


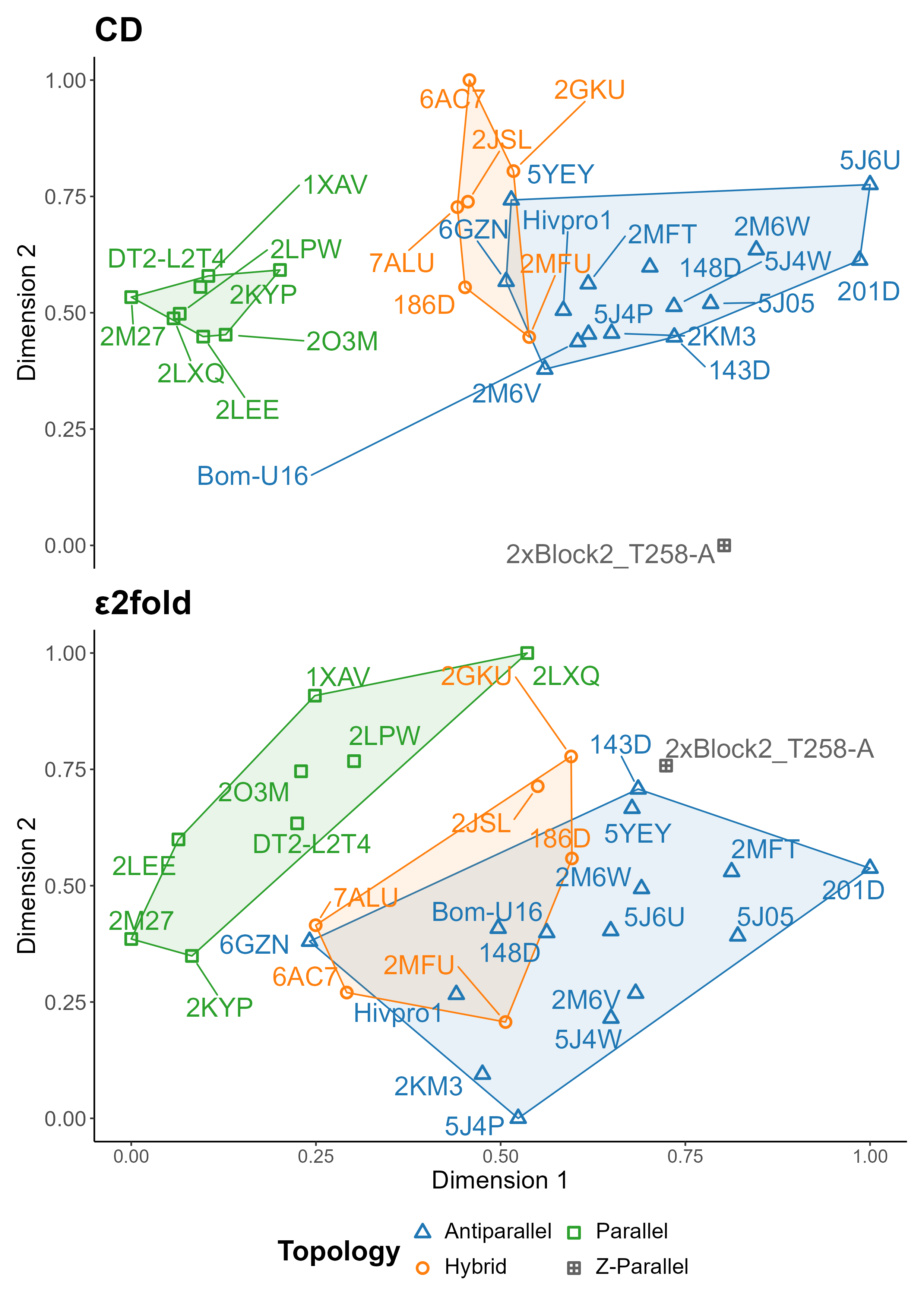


Figure S18. PCA results for CD (top) and ε2fold (bottom) visualized with topology groups.

### Groove type combination


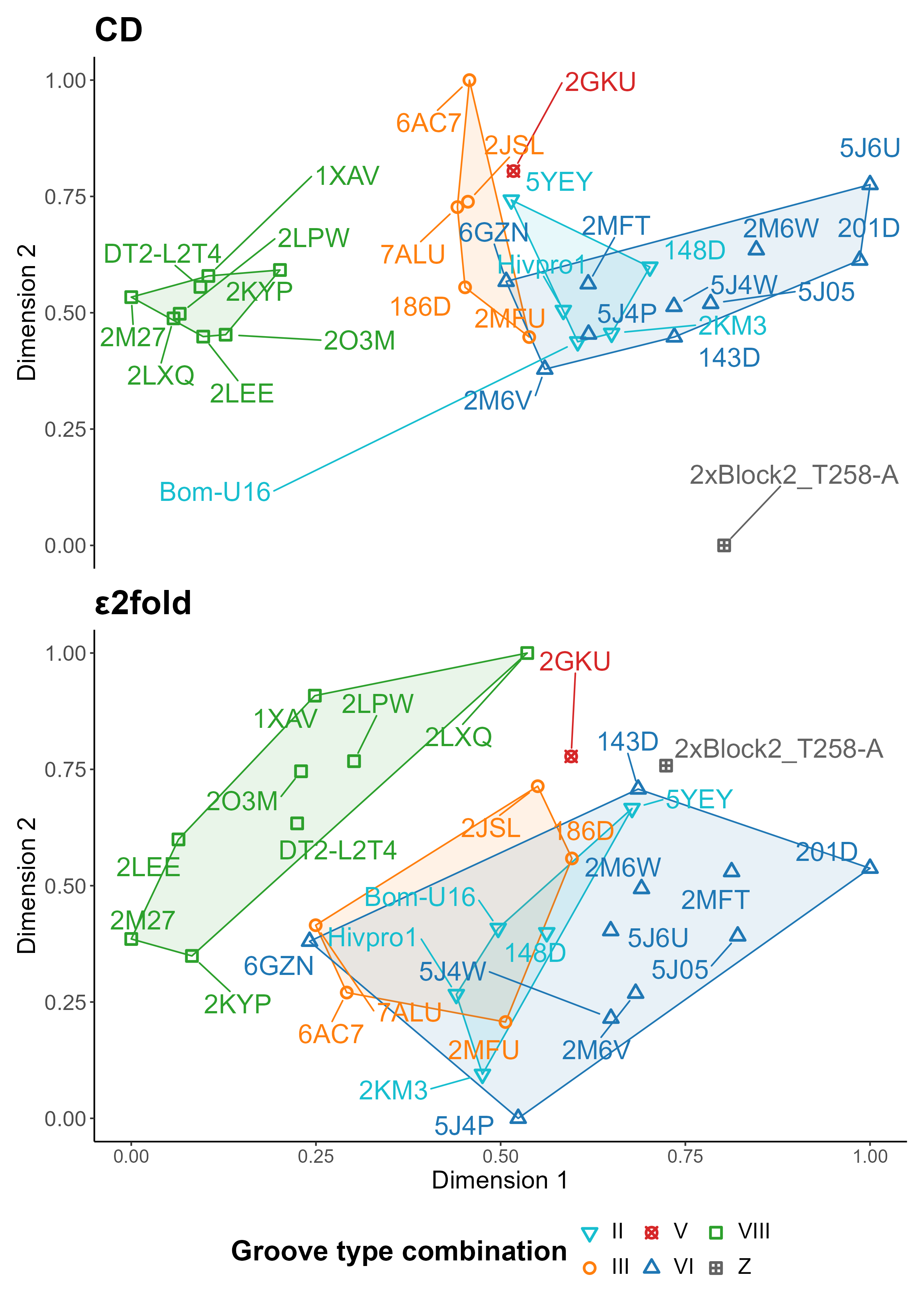


Figure S19. PCA results for CD (top) and ε2fold (bottom) visualized with groove type combination groups.

### Loop progression


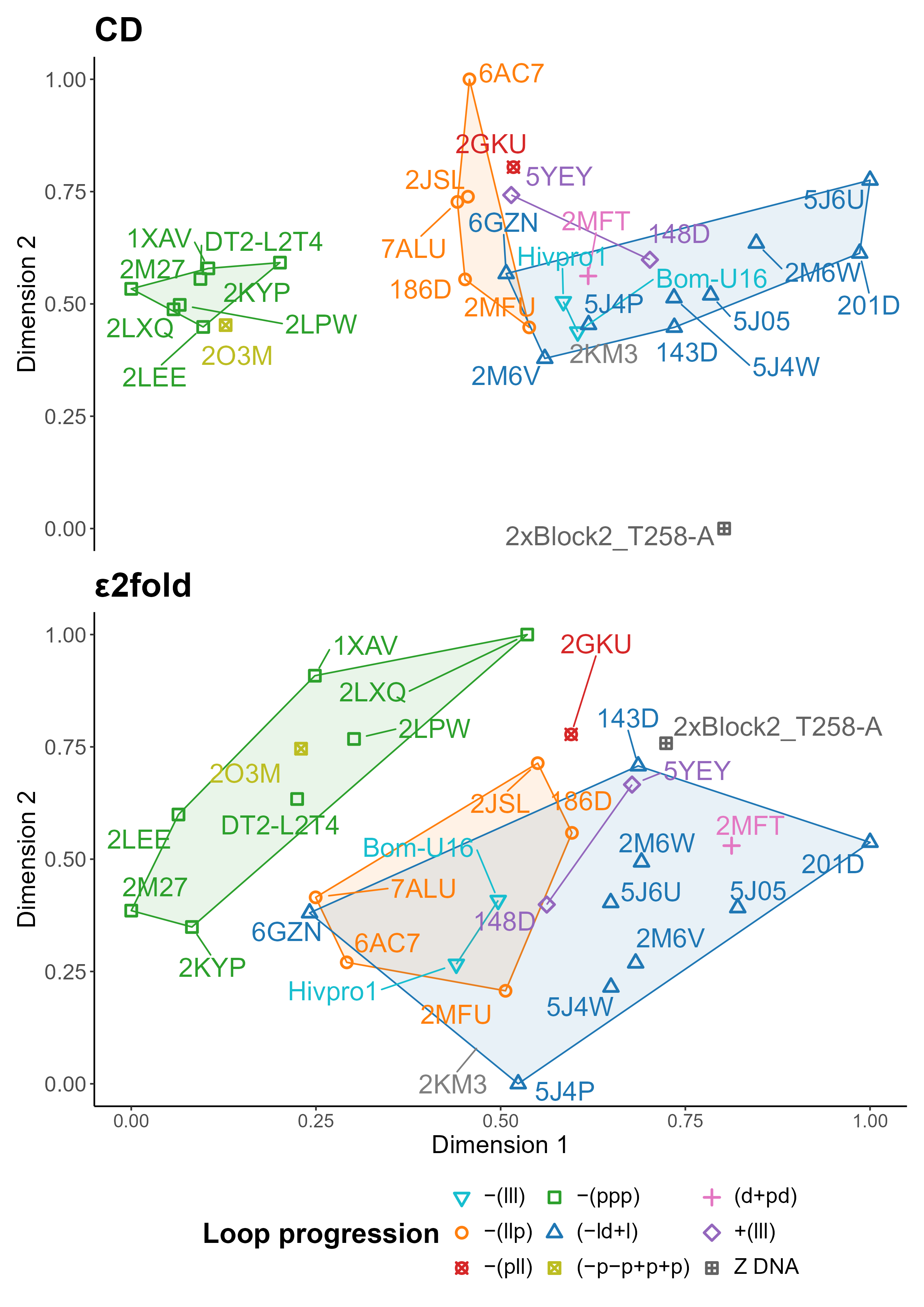


Figure S20. PCA results for CD (top) and ε2fold (bottom) visualized with loop progression groups.

### Tetrad x Loop progression


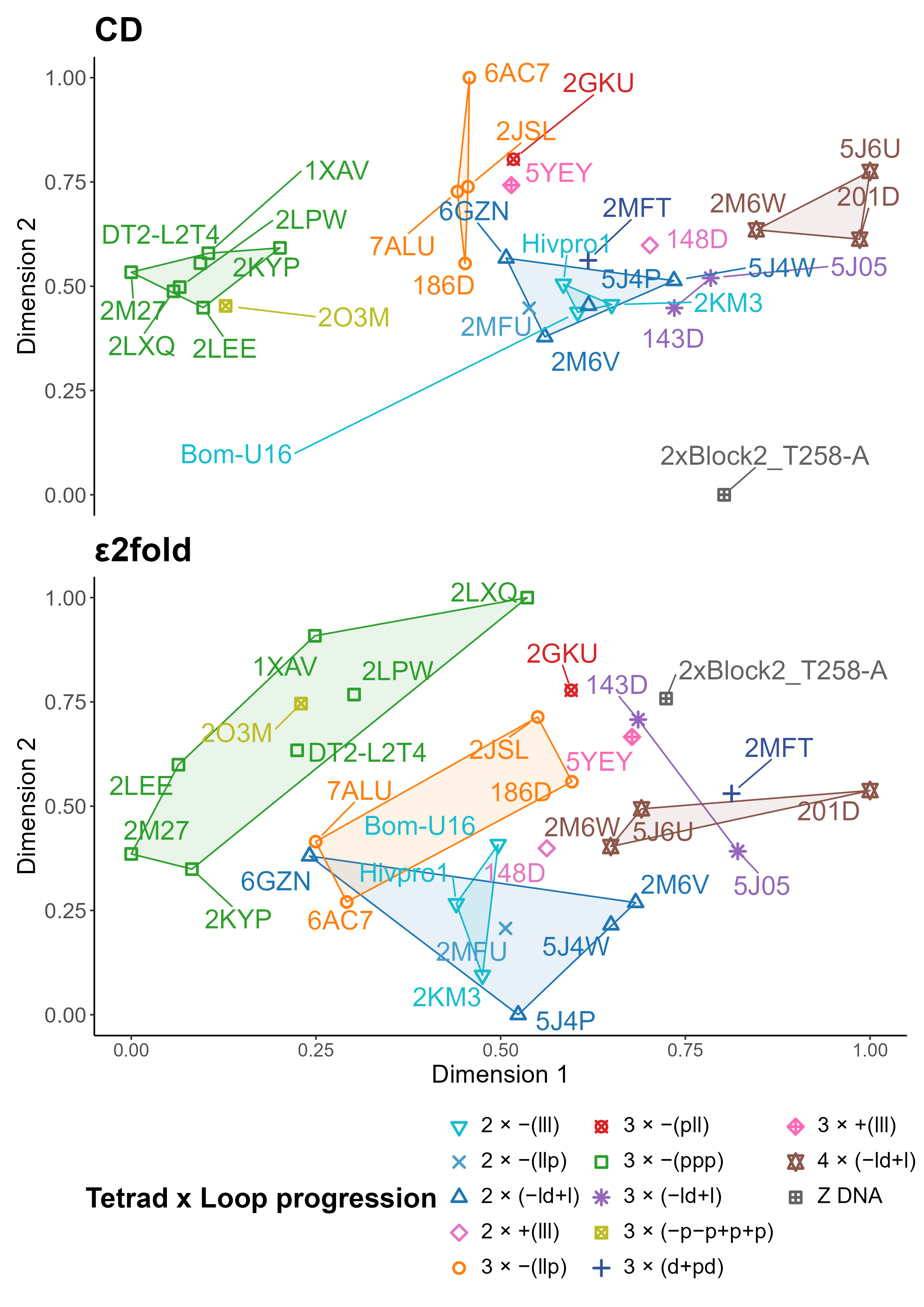


Figure S21. PCA results for CD (top) and ε2fold (bottom) visualized with tetrad x loop progression groups.

### GBA


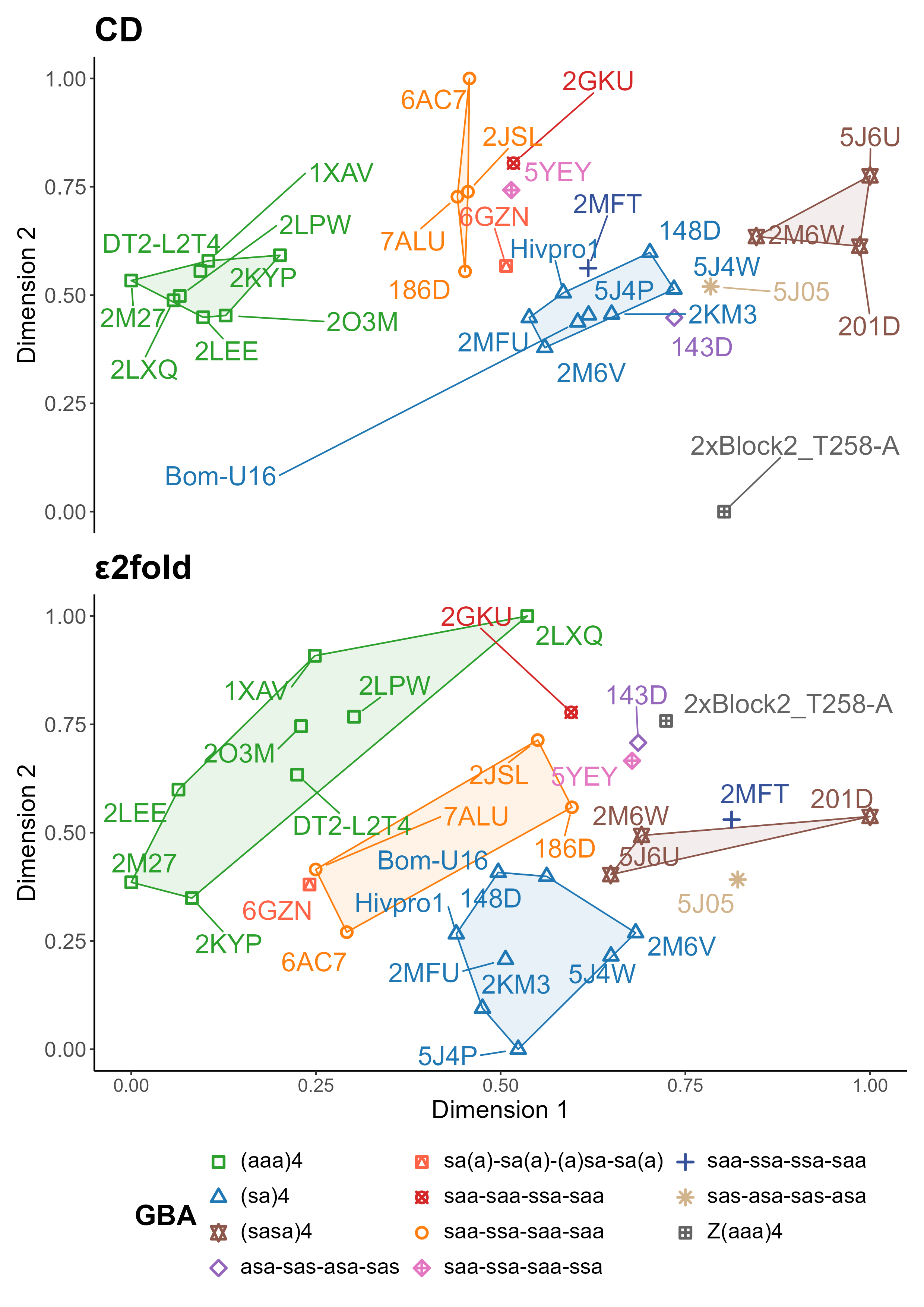


Figure S22. PCA results for CD (top) and ε2fold (bottom) visualized with GBA groups.

### Tetrad handedness


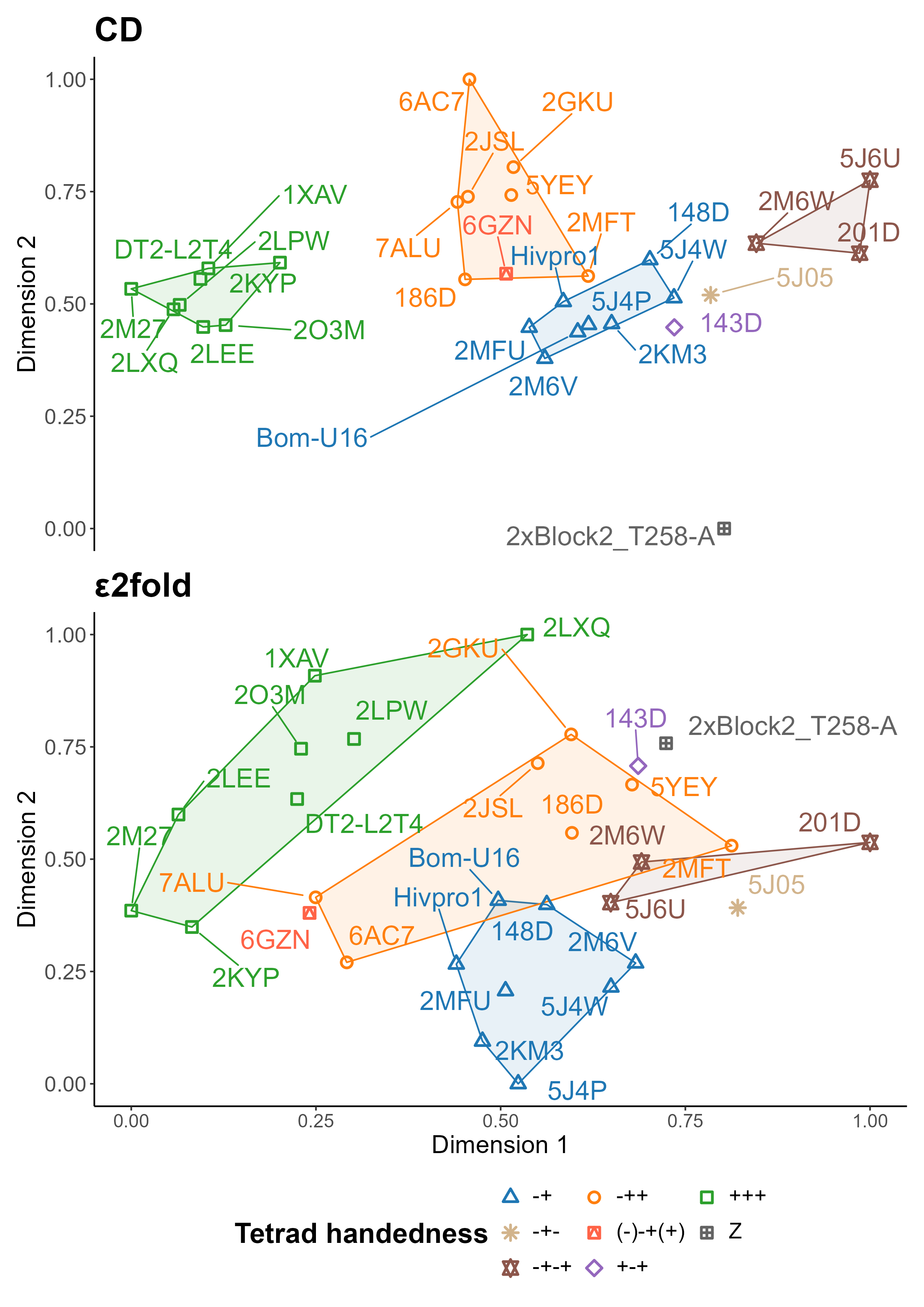


Figure S23. PCA results for CD (top) and ε2fold (bottom) visualized with tetrad handedness groups.

### GBA stacks


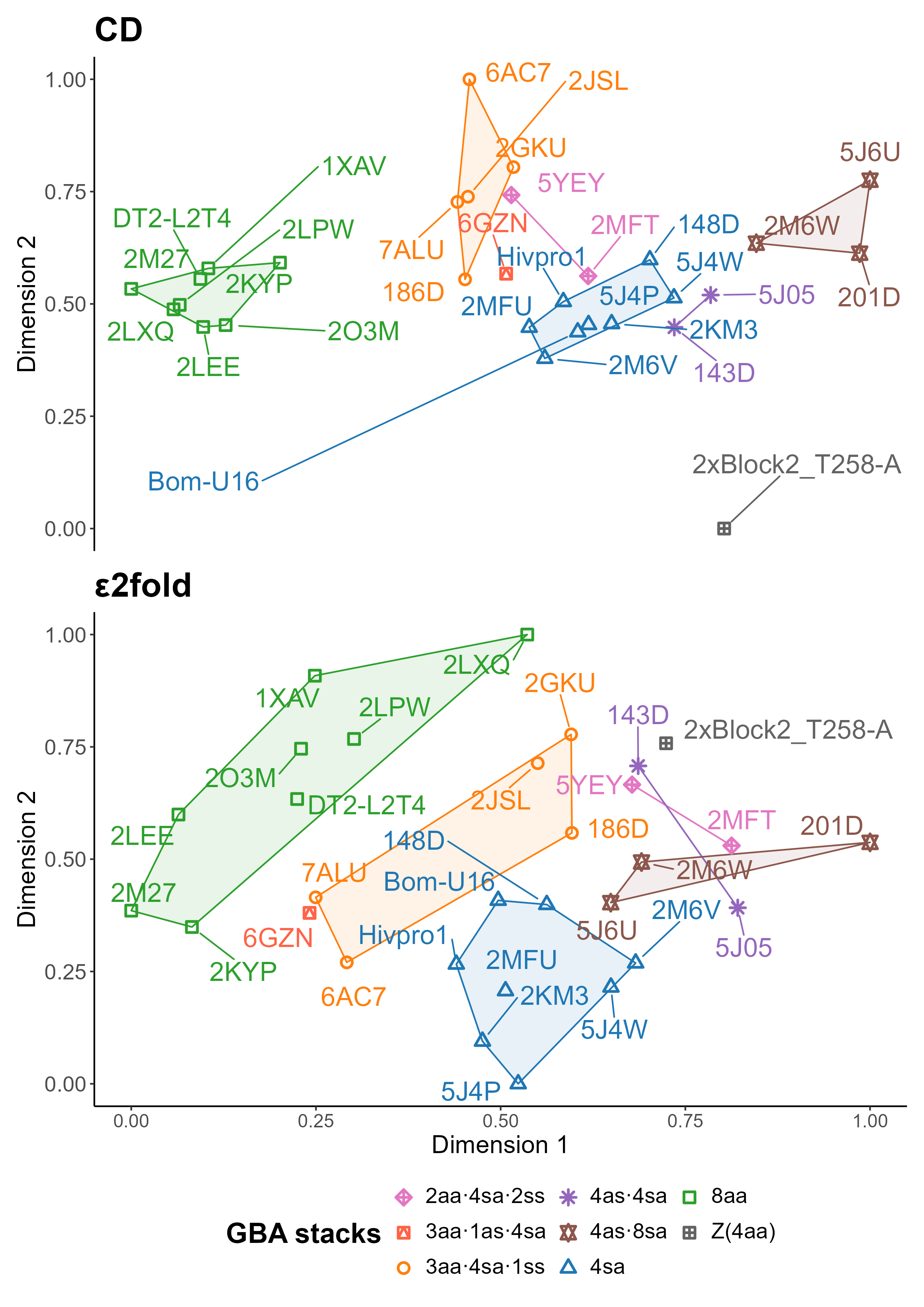


Figure S24. PCA results for CD (top) and ε2fold (bottom) visualized with GBA stack groups.

### GBA stacks with dimers


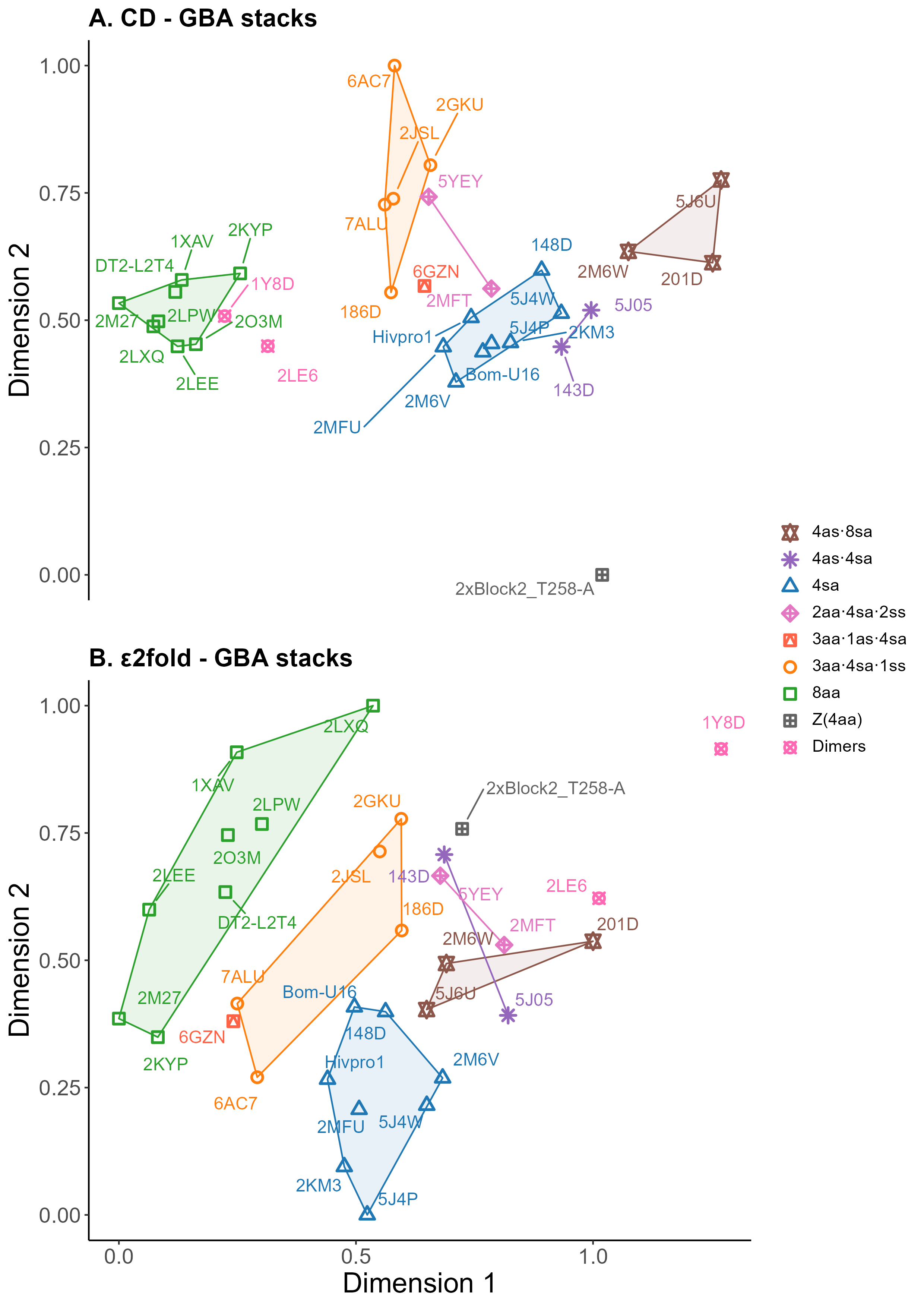


Figure S25. PCA results for CD (top) and ε2fold (bottom) visualized with GBA stack groups, onto which the signatures of 1Y8D and 2LE6 (pink) were mapped.
